# Single-cell omics and flow cytometry identify distinct immune states of dural and brain-infiltrating IL-17-producing γδ T cells after experimental stroke

**DOI:** 10.1186/s12974-026-03794-3

**Published:** 2026-04-09

**Authors:** Mingming Zha, Alina Jander, Haodi Cai, Marius Piepke, Karoline Degenhardt, Leo Winter, Tim Magnus, Mathias Gelderblom

**Affiliations:** 1 Department of Neurology, University Medical Center Hamburg-Eppendorf, Martinistrasse 52, 20246 Hamburg, Germany; 2 Department of Neurology, First Affiliated Hospital, School of Medicine, Zhejiang University, Zhejiang, China; 3 Department of Neurology, the First Affiliated Hospital of Soochow University, Jiangsu, China

**Keywords:** Acute ischemic stroke, γδ T cell, Dura, Meninges, ScRNAseq, Flow cytometry

## Abstract

**Background:**

γδ T cells boost inflammatory responses and exacerbate tissue damage after ischemic stroke. However, the origin, dynamics, and tissue adaptation of γδ T cells in the ischemic brain and its border regions remain poorly understood. A systematic integration of large-scale datasets is urgently needed. Here, we investigated the impact of ischemic stroke on the state of meningeal and brain-infiltrating γδ T cells and explored their potential contributions to post-stroke inflammation.

**Methods:**

We conducted an integrated analysis of publicly available single-cell RNA sequencing (scRNA-seq) datasets, which included meningeal and brain-infiltrating *Ptprc*^+^ (CD45^+^) immune cells following experimental stroke. γδ T cells were identified and subsequently classified into distinct subtypes through data integration and reference mapping. Subtype-specific functions, tissue residency signatures, migratory programs, and the cellular interactions between γδ T cells and endothelial cells or fibroblasts in the dura and brain were investigated, respectively. Key findings were validated by flow cytometry and immunofluorescence assays in vivo.

**Results:**

On day 2 post-experimental stroke, the number of parenchymal γδ T cells significantly increased while dural γδ T cells decreased. The majority of γδ T cells residing in the meninges and infiltrating the brain, both under homeostatic conditions and following stroke, were *Rorc*⁺ and belonged to the Vγ6⁺ γδ17 cell subset. Compared to dural γδ T cells, brain-infiltrating γδ T cells showed reduced tissue residency capacities, higher migratory pathway activation, and lower Ki‑67 positivity, indicating acute recruitment. In contrast, dural γδ T cells exhibited greater IL‑17-producing capacities on day 3. Redistributions of dural γδ T cells were analyzed, and immunofluorescence revealed a close spatial association between dural γδ T cells and CD31^+^ cells. Cell–cell communication analysis predicted increased interactions between γδ T cells and CD45^−^ cells in both the dura and the brain.

**Conclusion:**

Our data indicate that most meningeal and brain-infiltrating γδ T cells after stroke share an activated γδ17 phenotype but display compartmentalized dynamics in activation, proliferation, and migration. These results establish a foundation for further studies on the spatially distinct roles of γδ T cells in post-stroke immunity.

**Supplementary Information:**

The online version contains supplementary material available at 10.1186/s12974-026-03794-3.

## Introduction

Stroke is the third leading cause of mortality and long-term disability worldwide, with acute ischemic stroke accounting for the majority of cases [1, 2]. Beyond the vascular occlusion itself, inflammation and immunity critically shape the pathophysiology of ischemic brain injury [3]. Among the key players are γδ T cells, which are innate-like lymphocytes that rapidly respond to danger signals and represent the main source of interleukin-17A (IL-17A) in the ischemic brain. [4]. In the first hours and days after stroke, IL-17A promotes neutrophil recruitment into the injured parenchyma, thereby amplifying tissue damage [4, 5, 6]. Yet, the cellular origin and tissue-adaptation programs of these pathogenic γδ T cells remain poorly defined.

The meninges, composed of dura mater, arachnoid, and pia mater, form a protective interface between the brain, the skull, and the cerebrospinal fluid. Far from being a passive barrier, the dura harbors a diverse immune cell repertoire and has recently emerged as an active immunological hub [7, 8, 9, 10]. Direct anatomical connections among brain, dura, and bone marrow suggest that immune cells may traffic between these compartments [11, 12], with translational potential for therapeutic development such as drug delivery. Single-cell RNA sequencing (scRNAseq) has been widely used in stroke research [13], which enables researchers to explore rare but important cell populaions, such as γδ T cells. Under steady-state conditions, IL-17-producing γδ T cells are abundant in the meninges. These meningeal IL-17-producing γδ T cells have physiological effects on neurons and glial cells and modulate anxiety-like behavior and memory [14, 15]. Strikingly, both resident meningeal γδ T cells and brain-infiltrating γδ T cells after experimental stroke are enriched for Vγ6⁺ subsets and display robust IL-17A expression [15, 16]

These findings reveal a fundamental gap in our understanding. It is still unclear to what extent cerebral ischemia activates meningeal γδ T cells and how this activation contributes to the inflammatory cascade that drives secondary brain damage. Although multiple scRNAseq datasets of meningeal and brain immune compartments are available, an integrated analysis of γδ T cells across these tissues post-stroke is still absent. To address this knowledge gap, we performed a comprehensive integration of publicly available and in-house single-cell omics datasets. We then validated our findings experimentally using flow cytometry and immunofluorescence. The integrated approach enabled us to define the role of γδ T cells at the brain-meningeal interface in ischemic stroke and to propose directions for future research.

## Materials and methods

### Introduction to the publicly available scRNAseq datasets used in this study

Processed gene expression matrices of immune cells involving dura (sham and day 1 post-experimental stroke), pia (sham and day 1), and brain (sham, day 1, and day 3) were extracted from Beuker C et al. [17]. In brief, Beuker C et al. sorted brain- and border-associated CD45^+^ cells from C57BL/6 J male mice (10–16 weeks) undergoing transient middle cerebral artery occlusion (tMCAO) or sham operation at different time points following experimental stroke. And the scRNAseq data accession number was GSE189432 on the Gene Expression Omnibus (GEO) website.

Processed gene expression matrices of dural immune cells under sham, naïve, and day 3 post-tMCAO modeling conditions were extracted from Kolabas ZI et al. [18] and downloaded from the GEO website (access number: GSE192616). Kolabas ZI et al. arranged scRNAseq experiments on brain, meninges, calvaria, and other tissues to display molecular heterogeneity of bone marrow across the body post-experimental stroke. To reduce heterogeneity, brain scRNAseq data from Kolabas ZI et al. were not used for integration due to relatively higher mitochondria percentages.

Brain scRNAseq data at 3 h, 12 h, and 3 days post-tMCAO and sham status were extracted from Li et al. [19]. Processed gene expression matrix was not given, and the fastq documents were downloaded from the GEO database (accession number: PRJNA912889, PRJNA912890). Then the standard Cell Ranger (version 7.2.0) workflow was executed, and filtered output files were used for downstream analysis. Furthermore, processed gene expression matrices of brain scRNAseq data on sham and day 1 post-tMCAO modeling from Zheng et al. [13], and brain data at day 1 and day 2 post-tMCAO from Kim et al. [20] were also taken for integration. The accession numbers of the two aforementioned studies were GSE174574 and GSE197731, respectively.

Processed gene expression matrices of Vγ4 and Vγ6 γδ T cells from peripheral lymph nodes, ear skin, and thymus were extracted from Tan et al. [21], and the data accession number was GSE123400. Annotated Seurat object of γδ T cells from lymph nodes, lung, liver, small and large intestine, skin, and spleen were derived from du Halgouet et al. [22], and the dataset was downloaded from the GitHub link disclosed in the literature.

Data on sorted meningeal γδ T cells were extracted from Alves de Lima et al. [14], including cells from P7 and adult mice (accession number: GSE147262). Besides, dural CD45^−^ cells in homeostasis from Scheyltjens et al. [23] were used for cell–cell communication analysis with dural γδ T cells at different time points post-stroke, and the dataset was accessed from GSE191075.

We also did exploratory analysis on blood scRNAseq data from Lidia Garcia-Bonilla [24], containing data from sham, day 2, and day 14 post-tMCAO modeling, aiming to identify γδ T cells from the blood. The accessed number of scRNAseq data was GSE225948.

### Processing and analysis of the scRNAseq datasets

The scRNAseq data from the brain, pia, and dura [13, 17, 18, 19, 20] were integrated to characterize the impact of experimental stroke on the immune landscape surrounding the brain. In brief, the Seurat workflow was applied to each dataset [25]. Low-quality cells within each sample were removed based on total Unique Molecular Identifiers (nCount_RNA), gene counts (nFeature_RNA), and percentage of mitochondrial genes (detailed threshold for each sample was shown in the R codes in the Additional File 1). The DoubletFinder R package was used to identify doublets within each sample following the official guideline [26]. Mitochondrial and ribosomal RNA were removed from the gene expression matrix before clustering. Besides, we also deleted four genes (*Malat1*, *Gm26917*, *AY036118*, *Gm42418*) that might lead to bias during clustering, as mentioned in a previous study [27]. Variations attributable to differences in nCount_RNA, nFeature_RNA, and the immediate-early gene (Supplemental Table 1) signature [23] were regressed out before integration to reduce heterogeneity. Then, singlets were integrated with the harmony R package [28]. After clustering, canonical markers were used to label the cells. Non-immune cells, low-quality cells, and doublets were removed before downstream analysis. Of note, brain-associated endothelial cells and fibroblasts were extracted for further cell communication analysis with brain-infiltrating γδ T cells. For the immune cells from the brain and border regions, the harmony integration workflow was repeated to purify the dataset. Besides, all T cells, natural killer (NK) cells, and innate lymphoid cells (ILC) in the purified dataset were extracted and re-clustered to get finer annotations (αβ T cell, γδ T cell, ILC2, NK cell, and NKT cell). Then the finer annotations were transferred back to the integrated immune cell dataset and used for the following analysis pipelines (Supplemental Figs. 1 and 2).

This study also used different methods to annotate γδ T cells precisely. First, brain and border-region-associated γδ T cells from the integrated immune cell dataset mentioned above were extracted. Second, Vγ4 and Vγ6 γδ T cells from peripheral lymph nodes, ear skin, and thymus extracted from Tan et al. [21] were processed based on the quality control thresholds described in the literature and underwent additional doublet exclusion procedures. Third, an annotated Seurat object containing γδ T cells from lymph nodes, lung, liver, small and large intestine, skin, and spleen from du Halgouet et al. [22] was utilized directly. Fourth, sorted meningeal γδ T cells derived from Alves de Lima et al. [14] were processed with the standard Seurat workflow, and clusters contaminated with macrophages were identified based on the expression of *H2-Aa*, *Ly6d,* and *Cd79a* and removed from the dataset as recommended by the original study. Fifth, annotated scRNAseq data, including subtypes of T cells from baseline and day 3 post-stroke from in-house data, were utilized directly. And the workflow and details of the dataset process were shown below in the Generation of scRNASeq data part.

γδ T cells from these five resources were integrated with the harmony R package, and a similar integration pipeline was executed as mentioned above. Resolution was set to 3 to separate the cells well, and key markers derived from published studies were used to annotate the clusters [21, 22]. Characteristics of different clusters identified after integration were analyzed. To validate our annotations, we performed additional reference mapping steps (FindTransferAnchors and TransferData functions in the Seurat package) to annotate brain-infiltrating and meningeal γδ T cells, using the validated dataset from du Halgouet et al. [22] as a reference.

**Fig. 1:**
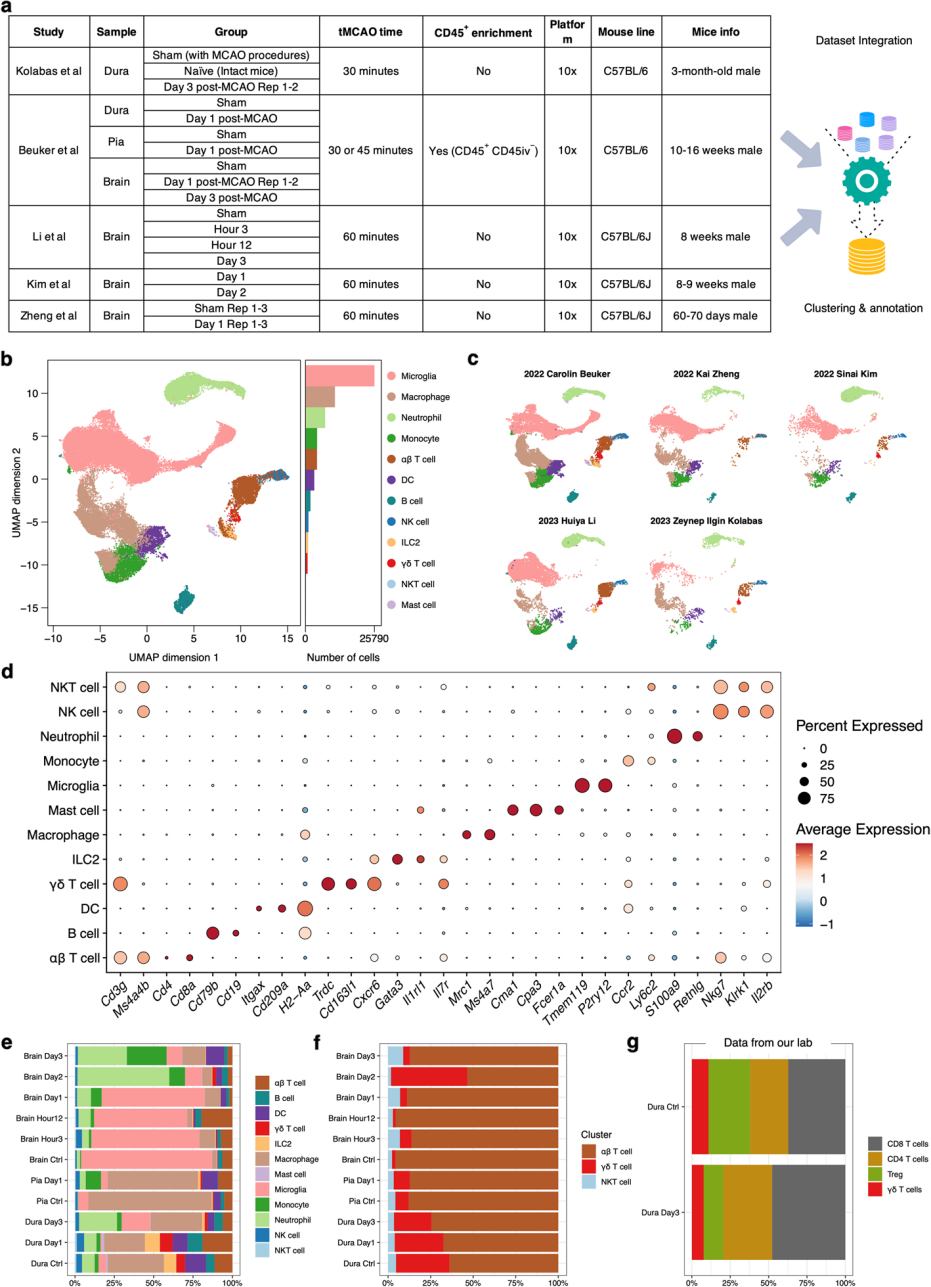
Analysis of single-cell sequencing data from the brain and border regions post-experimental stroke. **a**. Workflow for the integration of single-cell RNA sequencing (scRNAseq) data from five independent studies. **b**. Uniform Manifold Approximation and Projection (UMAP) plot of the integrated dataset, showing the distribution of major immune cell populations. **c**. UMAP visualization of the integrated scRNAseq dataset classified by different origins. **d**. Dot plot of canonical marker genes used for cell type annotation. Dot color indicates scaled average expression, and dot size represents the percentage of cells expressing the gene. **e**. Composition of major immune cell types across tissues and time points in the integrated scRNA-seq dataset. **f**. Proportion of γδ T cells among all T cells, plotted across tissues and time points. **g**. Frequency of γδ T cells among T cells in the dura at baseline and 3 days post-stroke in the in-house dataset. ScRNAseq was performed on flow-sorted dural leukocytes from C57BL/6 mice under homeostatic conditions and at day 3 after transient middle cerebral artery occlusion across two to three independent experimental replicates. T cells were subclustered to identify γδ T cells. Abbreviations: scRNAseq, single-cell RNA sequencing; UMAP, Uniform Manifold Approximation and Projection; tMCAO, transient middle cerebral artery occlusion; NK, natural killer; ILC, innate lymphoid cell; DC, dendritic cell; Treg, regulatory T cell

As for analysis of the blood scRNAseq data, the annotations from the original publication were used, and different samples were merged and normalized. T cells and NK cells (annotated as Tc and NK in the original datasets) were extracted. Then, reference mapping steps were arranged to annotate blood-derived T cells, using αβ T cell, γδ T cell, ILC2, NK cell, and NKT cell from Fig. 1 as the reference. The identified γδ T cells were mapped to the reference dataset from du Halgouet et al. to get their finer phenotypes. Percentages of different subtypes were compared at different time points post-experimental stroke.

### Differentially expressed gene identification and enrichment analysis

To characterize γδ T cells across different regions (dura, pia, and brain) and time points post-stroke, specific upregulated genes were identified using the ‘FindAllMarkers’ function in the Seurat R package. Subsequently, enrichment analysis with the clusterProfile R package [29] was conducted to elucidate the biological processes associated with the top-100 genes from each region/time point [30, 31, 32].

### Module score calculation

The γδ17 (*Il17a*, *Il22*, *Il23r*, *Adam12*, *Ptgs2*, *Dgat1*, *Cxcl10*, *Tmem176b*, *Aqp3*, *Ret*, *Cysltr1*, *Ltb4r1*, *Cd36*, *Ffar2*, *Serpinb1a*, and *Prdm1*) and IFN-γ gene signatures (*Ifng*, *Il2*, *Ccl3*, *Ccl4*, *Ccl5*, *Ccl9*, *Xcl1*, *Themis*, *Slamf6*, *Cd5*, *Cd6*, *Nkg7*, *Crtam*, *Lag3*, and *Cd160*) were extracted from Daniel Inácio et al. [33] and calculated for all subclusters of γδ T cells after integration using the ‘AddModuleScore’ function in Seurat [34]. The ‘AddModuleScore’ function calculates the average expression of a given signature per cell and subtracts it from the average expression of randomly selected control features [35]. Genes in the T cell activation involved in immune response gene ontology term from the Gene Ontology database (GO:0002286, gene list was shown in Supplemental Table 2), T cell proliferation term (GO:0042098, Supplemental Table 3), Tissue residency memory core residency signatures (Supplemental Table 4) extracted from Mackay LK et al., and T cell migration term (GO:0072678, Supplemental Table 5) were used for module score calculation for γδ T cells from brain and border regions [36].

### Analysis of the developmental potential of γδ T cells

The developmental potential of γδ T cells from the brain and border regions was analyzed with the CytoTRACE2 package [37]. The standard workflow following the official manual was performed, and ranks of developmental potential regarding tissue/group were displayed.

### Cell–cell communication analysis

Dural γδ T cells (stroke/control status; Beuker et al., Kolabas et al., and in-house data) were merged with dural CD45^−^ cells in homeostasis derived from Scheyltjens et al. [23]. Then, the merged Seurat object was log-normalized, and the CellChat R package [38] was utilized to explore potential cell–cell interactions between CD45^−^ cells (endothelial cells, epithelial cells, fibroblasts, pericytes) and γδ T cells in the dura at different time points post-experimental stroke. Moreover, we also investigated interactions between brain-infiltrating γδ T cells (extracted from Fig. 1) and brain-associated endothelial cells and fibroblasts (identified during the purification of Fig. 1-related data) to reveal potential infiltration pathways for brain-associated γδ T cells.

### Visualization of scRNAseq results

The entire analytical workflow for scRNAseq data was conducted in R [39]. The scRNAtoolVis R package on the GitHub platform was used to create the Uniform Manifold Approximation and Projection (UMAP) plot and volcano plot [40]. The Scillus R package on the GitHub platform was used to create percentage stack plots and display enrichment analysis results [41].

### Animals

All animal experiments were performed following the regulations of the national and animal facility of the University Medical Center Hamburg-Eppendorf and were approved by the local animal care committee. Information on all mice used in this study is shown in Additional File 2. The *Tcrδ*-GDL mouse strain was used for the majority of flow cytometric experiments in this study, and littermates were randomly allocated to different groups. Both genders were utilized, considering sex differences. For Ki-67 staining, three C57BL6 mice on day 3 post-stroke were used. The *Tcrδ*-H2BEGFP mouse strain was used for immunofluorescence analysis in this study. Detailed introductions on these mouse strains have been thoroughly described in previous publications [42, 43]. In brief, cytoplasmic enhanced green fluorescence protein (eGFP) expression signal in the *Tcrδ*-GDL mice can be used to identify γδ T cells. The fusion of human histone H2B with eGFP preserves the signal of the reporter protein after paraformaldehyde fixation in *Tcrδ*-H2BEGFP mice.

### Stroke modeling

The transient middle cerebral artery occlusion stroke modeling was conducted by experienced technicians who were blinded to group allocations, and detailed modeling methods were described in previous publications with minor changes [6]. In brief, mice were anesthetized with Isoflurane (Sedaconda, Cat# 7,002,244.00.00, 1% to 2% v/v oxygen). Buprenorphine (Bayer, Cat# 401,513.00.00, 0.03 mg/kg body weight) was injected intraperitoneally for analgesia. An intraluminal filament (Doccol, Cat# 602312PK10Re) was inserted into the left middle cerebral artery for 45 min and then removed. After modeling, mice were placed on a heating pad with facilitated access to wet food and water. Experienced caregivers visited and attended to these mice at least twice a day. The alterations in weight, spontaneous behaviors, and facial expressions were recorded.

Brain infarction was determined using animal magnetic resonance imaging (MRI) provided by the Department of Diagnostic and Interventional Radiology and Nuclear Medicine in University Medical Center Hamburg Eppendorf (ClinScan, Bruker). Two Mice in the day 1 and 3 groups did not undergo the MRI examination. Confirmation of stroke was based on the observation of stroke-related behavioral deficits and visual inspection of infarcts during the flow cytometric experiments.

### Tissue processing

Mice were euthanized with CO_2_/O_2_ and transcardially perfused with ice-cold 1 × phosphate-buffered saline (PBS, [Gibco, Cat# 10010023], 9.6 ml/min, 3 min). The neck was exposed, and cervical lymph nodes were dissected, mechanically dissociated by grinding, and then filtered through a 40-µm cell strainer to obtain a single-cell suspension. Skulls with dural meninges were isolated from the skull base by cutting in a transverse plane on both sides. The dura was extracted from the skull under a binocular microscope [9, 23, 44], cut into pieces, and digested with a digestion mix (Dulbecco's Modified Eagle Medium [DMEM, Gibco, Cat#41965039] + 2.0 mg/ml Collagenase [Sigma, Cat# C2139] + 0.2 mg/ml DNase [Roche, Cat# 11284932001]) for 15 min with agitation. Digested dura was filtered through a 40 µm cell strainer after grinding. Brains were extracted and divided into ipsilateral (left) and contralateral (right) sides. For each side, the brain was cut into pieces and digested (digestion mix: 1 mg/ml collagenase and 0.1 mg/ml DNase I in DMEM) for 30 min with agitation. Then the digested brain was transferred through a 40 µm cell strainer after grinding.

After centrifugation, a standard erythrocyte lysis step was performed (Biolegend, Cat# 422,401). Discontinuous Percoll gradients were used for brain cell isolation. Isotonic Percoll solution was prepared (9:1 Percoll [Cytiva, Cat# 10341908] in 10 × PBS [Sigma Aldrich, Cat# D1408-500ML]). For each hemisphere, the pellet after centrifugation was resuspended in 2.8 ml Percoll B solution (1:2, isotonic Percoll solution in DMEM), and 2.8 ml Percoll A solution (4.7:1.3, isotonic Percoll solution in 1 × PBS) was placed beneath the Percoll B layer. The parameter of centrifugation was set as 1350 g, 30 min, 4℃, accelerations off, and brake off. After that, 2 ml of cloudy middle-layer fluid was extracted and washed with 1 × PBS before further staining. Then the cells were centrifuged and used for subsequent staining.

### Immunofluorescence

We arranged immunofluorescence experiments to show the distributions of γδ T cells in homeostasis. The skull with dural meninges from sham *Tcrδ*-H2BEGFP mice was isolated as mentioned above, fixed in 2% paraformaldehyde solution for 10 min, and transferred into 1 × PBS overnight. Then the whole mount dura was extracted from the skull under a binocular microscope and blocked with blocking solution (2% BSA [Thermofisher, Cat# A34785] and 5% v/v Goat serum [Biozol, Cat# LIN-ENG9010] in PBS-Tween buffer [0.1%, Merck, Cat# P7949-100ML]) for 1 h at room temperature. The dura was incubated with primary antibodies (Armenian Hamster-anti-mouse CD3ε, Biolegend, Cat# 100,302; Rat-anti-mouse CD31, dianova, Cat# DIA-310) at 4 °C overnight and washed with PBS-Tween (PBS-T) buffer for 5 min. After washing 6 times, the dura was incubated with secondary antibodies (Goat-anti-Armenian Hamster Alexa Fluor™ 647, Invitrogen, Cat# A78967; Goat-anti-rat Alexa Fluor™ 555, Invitrogen, Cat# A21434) at 4 °C overnight and washed with PBS-Tween buffer 7 times. Then the dura was flattened on a glass slide. The mounting media (ROTH, Cat# HP20.1) was applied, and a coverslip was placed on the dura. The slide was checked under the Leica SP8 confocal microscope after drying overnight.

To investigate interactions between dural γδ T cells and CD45^−^ cells, whole mount dura mater was extracted from *Tcrδ*-H2BEGFP mice three days after tMCAO and blocked with 5% donkey serum in 1% in 0.05% PBS-T for one hour at room temperature. The tissue was then incubated with the following primary antibodies overnight at 4 °C: Rat-anti-mouse CD11a (1:100, Biolegend, Cat# 162,902), goat-anti-mouse ICAM2 (1:30, Invitrogen, Cat# PA5-47,939), rabbit-anti-mouse-Laminin (1:1000, Sigma, Cat# L9393). Washing was performed three times with 0.05% PBS-T for 5 min at room temperature before secondary antibodies (donkey-anti-rat-IgG AF555 Abcam, Cat# ab150154; donkey-anti-goat-IgG AF647, Invitrogen, Cat# A-21447; Donkey-anti-rabbit-IgG AF405, abcam, Cat# ab175651) were incubated for 60 min at room temperature, protected from light. Following a second washing step, whole mount meninges were placed on a glass slide and embedded in mounting medium (ProLong™ Gold Antifade Mountant, Invitrogen, Cat# P10144). Representative images of sinus and parasinus regions were taken on a Leica SP8 confocal microscope.

### Flow cytometric experiments

Detailed methodologies of flow cytometric experiments were modified based on previous publications [5, 45]. In brief, single-cell solutions derived from dura, brain, and lymph nodes were washed with 1 × PBS. Dead cells were labeled either with LIVE/DEAD™ Fixable Near-IR Dead Cell Stain Kit (Invitrogen™ # L34975) or Zombie Red™ (BioLegend Cat# 423,110) live-dead staining kit for 15 (Zombie Red™) or 30 (Invitrogen™ # L34975) minutes on ice. Fc blocker (BioXCell, Cat# 5806/0715) was added to block non-specific binding. Cells were incubated with fluorescent antibodies on ice for 30 min, protected from light. Total counts of γδ T cells were analyzed with the BD Trucount™ Absolute Counting Tubes (BD Biosciences Cat# 340,334). To analyze intracellular IL-17A or Ki-67 levels, stimulation buffer including Brefeldin A (3 μg/ml, Invitrogen, Cat# 00–450651), Phorbol 12-Myristate 13-Acetate (100 ng/ml, Sigma, Cat# P1585-1MG), and Ionomycin (1 μg/ml, Sigma, Cat# I0634-1MG) was used to stimulate γδ T cells for 4 h in the 37 °C incubator [45]. After stimulation, cells were fixed and permeabilized before intracellular staining with the True Nuclear Fix, Nuclear Staining Buffer Kit (Biolegend, Cat# 424,401).

Four different flow cytometric panels were established in this study. To quantify γδ T cells at baseline and on days 1, 2, and 3 after experimental stroke, the following antibody panel was used for flow cytometry: CD45 (AF700, clone: 30-F11, Thermo Fisher Scientific Cat# 56–0451-82, RRID:AB_891454), CD3ε (PE, clone: 145-2C11, BioLegend Cat# 100,308, RRID:AB_312673), NK1.1 (BUV737, clone: PK136, BD Biosciences Cat# 741,715, RRID:AB_2871088), TCRγ/δ (BV421, clone: GL3, BioLegend Cat# 118,119, RRID:AB_10896753), CD4 (PerCP, clone: GK 1.5, BioLegend Cat# 100,432, RRID:AB_893323), CD8a (BUV395, clone: 53–6.7, BD Biosciences Cat# 563,786, RRID: AB_2732919), CCR6 (BV510/480, clone: 140,706, BD Biosciences Cat# 747,832, RRID:AB_2872295), CD44 (PE-Cy7, clone: IM7, BioLegend Cat# 103,030, RRID:AB_830787), and CD69 (BV785, clone: H1.2F3, BioLegend Cat# 104,543, RRID:AB_2629640). Gating thresholds for CCR6, CD44, and CD69 were based on the staining of γδ T cells from lymph nodes in each experiment.

The following antibody panel was used to stimulate γδ T cells isolated from the dura mater (baseline and day 3 post-stroke) and the infarcted brain (day 3 post-stroke): CD45 (BUV563, clone: 30-F11, BD Biosciences Cat# 612,924, RRID:AB_2870209), CD11b (PerCP, clone: M1/70, BioLegend Cat# 101,230, RRID:AB_2129374), B220 (BUV805, clone: RA3-6B2, BD Biosciences Cat# 748,867, RRID:AB_2873270), CD3 (BUV737, clone: 17A2, BD Biosciences Cat# 612,803, RRID:AB_2870130), TCRγ/δ (BV421, clone: GL3, BioLegend Cat# 118,119, RRID:AB_10896753), and IL-17A (PE, clone: eBio17B7, Thermo Fisher Scientific Cat# 12–7177-81, RRID:AB_763582). The isotype control (PE, clone: eBR2a, Thermo Fisher Scientific Cat# 12–4321-80, RRID:AB_1834380) was used to determine the threshold for IL-17A.

To quantify the Ki-67 expression (dura, infarcted brain and cervical lymph nodes at day 3 post-experimental stroke), the following antibodies were utilized: CD45 (BV510, clone: 30-F11, BioLegend Cat# 103,137, RRID:AB_2561392), CD3 (BUV395, clone: 17A2, BD Biosciences Cat# 740,268, RRID:AB_2687927), B220 (BV570, clone: RA3-6B2, BioLegend Cat# 103,237, RRID:AB_10900264), NK1.1 (BV711, clone: PK136, BioLegend Cat# 108,745, RRID:AB_2563286), TCR γ/δ (FITC, clone: GL3, BioLegend Cat# 118,106, RRID:AB_313830) and Ki-67 (BUV563, clone: SolA15, Invitrogen #365–5698-82, RRID:AB_2925398).

The surface expression of migration-related molecules on γδ T cells in the dura (baseline and 3 days post-stroke) and infarcted brain at 3 days post-stroke was evaluated by flow cytometry using the following antibody panel: CD45 (Percp, clone: 30-F11, Biolegend Cat# 103,130, RRID:AB_893339), CD11b (PE-Cy7, clone: M1/70, Biolegend Cat# 101,216, RRID:AB_312799), B220 (BUV805, clone: RA3-6B2, BD Biosciences Cat# 748,867, RRID:AB_2873270), CD3 (BUV395, clone: 17A2, BD Biosciences Cat# 740,268, RRID:AB_2687927), TCRγ/δ (BV605, clone: GL3, Biolegend Cat# 118,129, RRID:AB_2563356), CD11a (APC, clone: I21/7, Biolegend Cat# 153,110, RRID:AB_2716219), CXCR3 (BV421, clone: CXCR3-173, Biolegend Cat# 126,521, RRID:AB_10900974), and CXCR4 (PE, clone: L276F12, Biolegend Cat# 146,506, RRID:AB_2562783). Corresponding isotype controls (Biolegend Cat# 400,511, Cat# 400,935, and Cat# 401,208) from each experiment were used to set thresholds for CD11a, CXCR3, and CXCR4 positive cells, respectively. All measurements of the flow cytometric experiments were finished on the BD FACS Symphony A3.

### Generation of scRNASeq data

Four male C57BL/6 mice were pooled per condition (control and day 3 post-experimental stroke), with two to three independent replicates per condition. To minimize age-related variability, all mice were 22–26 weeks old at the time of sacrifice. Meningeal cells were isolated, as described above, and labeled with TotalSeq™-C hashtag antibodies (1–3; clones M1/42 and 30-F11; BioLegend; Cat. No. 155861, 155,863, 155,865) under the respective conditions. Hashtag antibodies and the TotalSeq™-C Mouse Universal Cocktail v1.0 (BioLegend; Cat. No. 199903) were prepared according to the manufacturer’s instructions.

Briefly, isolated meningeal cells were incubated with Fc blocker (anti-mouse CD16/CD32, 1:100) for 10 min at 4 °C, followed by incubation with TotalSeq™-C hashtag antibodies (1 ng/µl) for 10 min on ice. Cells were subsequently stained with the TotalSeq™-C cocktail, including an anti-CD45 antibody (1:200; clone I3/2.3, Biolegend, Cat. No. 147707), for 30 min at 4 °C protected from light. After three washes with cell staining buffer (BioLegend; Cat. No. 420201), cells were resuspended in PBS supplemented with 2% FBS. Propidium iodide (1:1000, Sigma Aldrich, Cat. No. P4864) was added 5 min before sorting for live/dead staining. Live CD45^hi^ meningeal leukocytes were sorted by fluorescence-activated cell sorting on a BD Aria Fusion using a 100-µm nozzle and collected in 3% FBS in PBS in pre-coated 1.5-ml LoBind® tubes.

A total of 40,000 meningeal leukocytes were loaded on a Chromium Next GEM Chip G (10 × Genomics) using the Chromium Next GEM Single Cell 5’ Reagent kit v1.1 to generate single-cell gel beads-in-emulsion (GEM). Gene expression (GEX) and cell surface protein (CSP) libraries were prepared according to the manufacturer’s instructions. Briefly, following GEM generation and reverse transcription, GEMs were broken, and cDNA derived from poly-adenylated mRNA and DNA from CSP Feature Barcode were purified and amplified. Amplified full-length cDNA from poly-adenylated mRNA was enzymatically fragmentated and subjected to end Repair, A-tailing, adaptor ligation, and sample index PCR to generate GEX libraries. CSP libraries were constructed from amplified Feature Barcode products by sample index PCR, incorporating Illumina sequencing adapters and dual indices. Final dual-indexed libraries were sequenced on an Illumina NovaSeq X Plus system (Novogene) with an average depth of 30,000 reads per cell for GEX libraries and 5,000 reads per cell for CSP libraries.

Sequencing reads were processed with Cell Ranger (v. 7.2.0, 10X Genomics). Ambient RNA and cell doublets were removed using CellBender v 0.3.0 [46] and Solo v. 0.6 [47], respectively. Cells were further filtered based on total UMI counts, number of detected genes, and the proportion of mitochondrial gene expression. Hashtag-based demultiplexing was then performed on filtered cells using the *solo* hash-solo module. All downstream analysis steps were conducted in R (v. 4.3.0). The GEX data of each demultiplexed sample were normalized using the SCTransform function in Seurat [25]. CSP data was normalized using the centered log ratio transformation. After principal component analysis and data integration with the Harmony R package [28], the first 12 components of the harmony-corrected embedding were used for neighborhood graph construction, followed by Leiden clustering with a resolution of 0.4. The resulting clusters were manually annotated based on their cluster markers, obtained with Seurat’s FindAllMarkers function, and corresponding CSP expression patterns. The analyses of this part were performed by the Bioinformatic Core Facility in the University Medical Center Hamburg-Eppendorf.

### Statistical analysis

Analysis of the flow cytometric data was performed using FlowJo software (BD, version 10.10). Results of flow cytometric experiments were processed by GraphPad Prism (version: 10.2.3). Normality (Shapiro–Wilk (W) test) and homogeneity of variance (Brown-Forsythe test) of the data were checked before significance analyses. For comparisons among more than two groups, the analysis of variance (ANOVA), the Brown-Forsythe and Welch ANOVA test, or the Kruskal–Wallis test with multiple comparisons test was used based on the results of normality and homogeneity of variance tests. For comparison between the two groups, the unpaired t-test, the unpaired t-test with Welch’s correction, the Mann–Whitney test, the paired t-test, or the Wilcoxon matched-pairs signed rank test was applied as appropriate. A *P*-value of < 0.05 was considered significant for all tests.

## Results

### Dural and brain-infiltrating γδ T cell subsets show dynamic changes following tMCAO

A total of 61,245 *Ptprc*^+^ cells were identified after integrating scRNAseq datasets involving immune cells from dura, pia, or brain at sham/naïve status, 3 h, 12 h, 1 day, 2 days, or 3 days post-experimental stroke (Fig. 1a-c). Canonical markers used to label cells are displayed in Fig. 1d, and *Trdc*, *Cd163l1*, and *Cxcr6* were used to annotate γδ T cells. Percentages of different immune cell subpopulations were calculated for each tissue and time point, respectively (Fig. 1e). The proportion of dural γδ T cells decreased by day 3 post-stroke compared to baseline and day 1 (Fig. 1e-f), and this trend was confirmed in the dataset generated from our lab (Fig. 1g). In contrast, the percentage of γδ T cells in the ischemic brain was elevated relative to baseline, especially on day 2 (Fig. 1e-f).

**Fig. 2:**
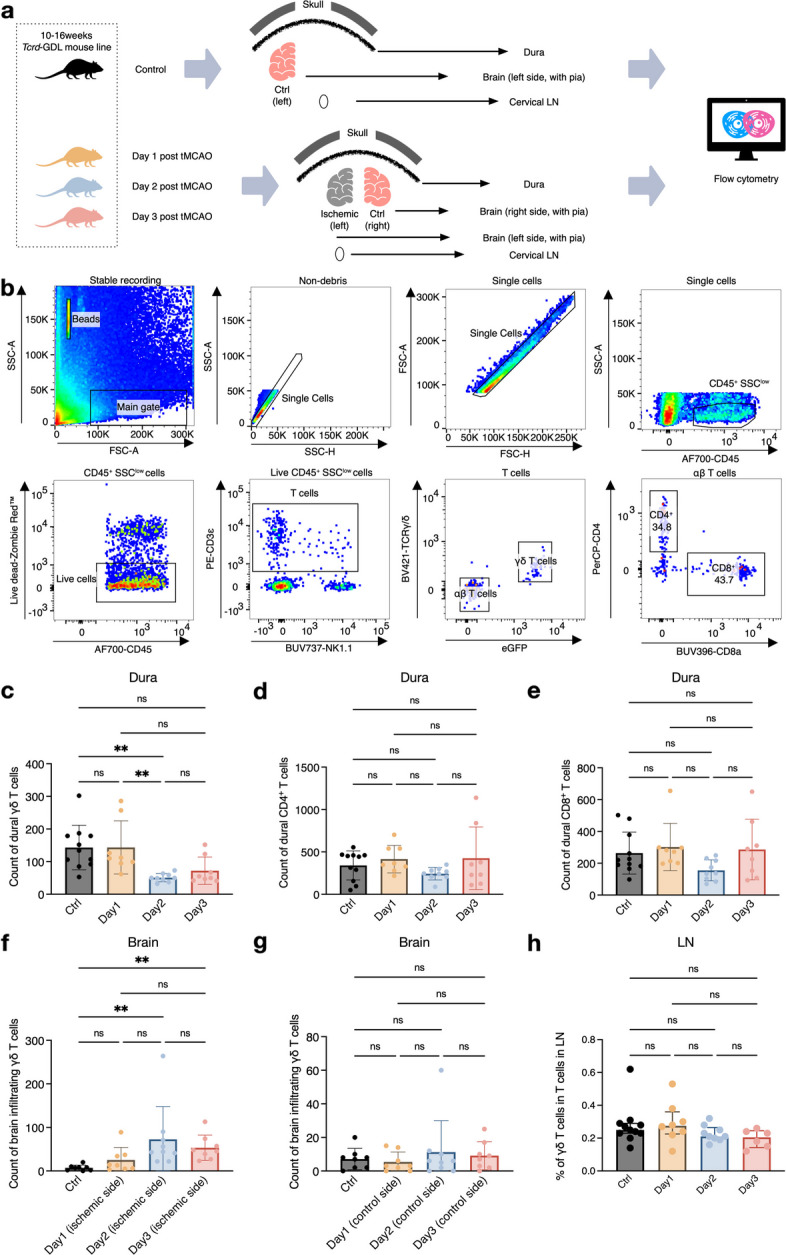
Divergent​ dynamics of γδ T cell abundance are seen between the dura and parenchyma. **a**. Schematic overview of the experimental design for flow cytometric analysis. **b**. Representative gating strategy for identification of CD4⁺, CD8⁺, and γδ T cells. Cell counts were determined using BD Trucount™ Absolute Counting Tubes. **c**. Absolute counts of dural γδ T cells were significantly reduced on day 2 post-stroke compared to sham controls and day 1. **d**-**e**. Absolute counts of CD4⁺ (**d**) and CD8⁺ T cells (**e**) showed no significant differences across groups. **f**. Absolute counts of γδ T cells in the ipsilateral brain were significantly increased on days 2 and 3 post-stroke compared to controls.​ **g**. No significant difference in γδ T cell counts was observed among naïve controls and the contralateral hemisphere at any time point.​ **h**. Proportions of γδ T cells among total T cells in cervical LNs were similar across all groups. For c-h, data are presented as mean ± standard deviation; *n* = 8–11 *Tcrδ*-GDL mice per group across 3–4 independent experiments per time point. For cervical lymph nodes (LN) analyses, two samples at day 3 were excluded due to low event counts. Significance was determined by ANOVA or the Kruskal–Wallis test with appropriate multiple comparisons. *P < 0.05, **P < 0.01, ***P < 0.001.​ Abbreviations: tMCAO, transient middle cerebral artery occlusion; LN, lymph nodes; FSC, forward scatter; SSC, side scatter; eGFP, enhanced green fluorescence protein; ANOVA, analysis of variance; ns, non-significant

To validate the findings in the scRNAseq analysis, we analyzed proportions of T cells in the dura, brain, and cervical lymph nodes at days 1, 2, and 3 after experimental stroke by flow cytometry (Fig. 2a). A detailed gating strategy is shown in Fig. 2b. The absolute count of dural γδ T cells was significantly lower in the day 2 group compared with the baseline and day 1 group. (Fig. 2c). Meanwhile, dural CD4^+^ and CD8^+^ T cells remained similar among different time points (Fig. 2d-e).

In contrast, the number of γδ T cells in the ischemic brain was significantly higher on days 2 and 3 post-stroke (Fig. 2f), indicating substantial infiltration during stroke progression. This trend was in accordance with the findings in Fig. 1. Of note, the ischemic insult had no measurable effect on the absolute count of γδ T cells in the contralateral hemisphere (Fig. 2g). Furthermore, the relative frequency of γδ T cells (among viable T cells) in cervical lymph nodes was similar regardless of time points post-stroke (Fig. 2h).

In summary, we observed divergent γδ T cell dynamics between meningeal and parenchymal compartments after stroke, characterized by a decrease in the dura versus an increase in the ischemic brain parenchyma.

### Rorc^+^ Il17a^+^ γδ T cells are the major subset in homeostasis and during stroke progression in the brain and border regions

**Fig. 3:**
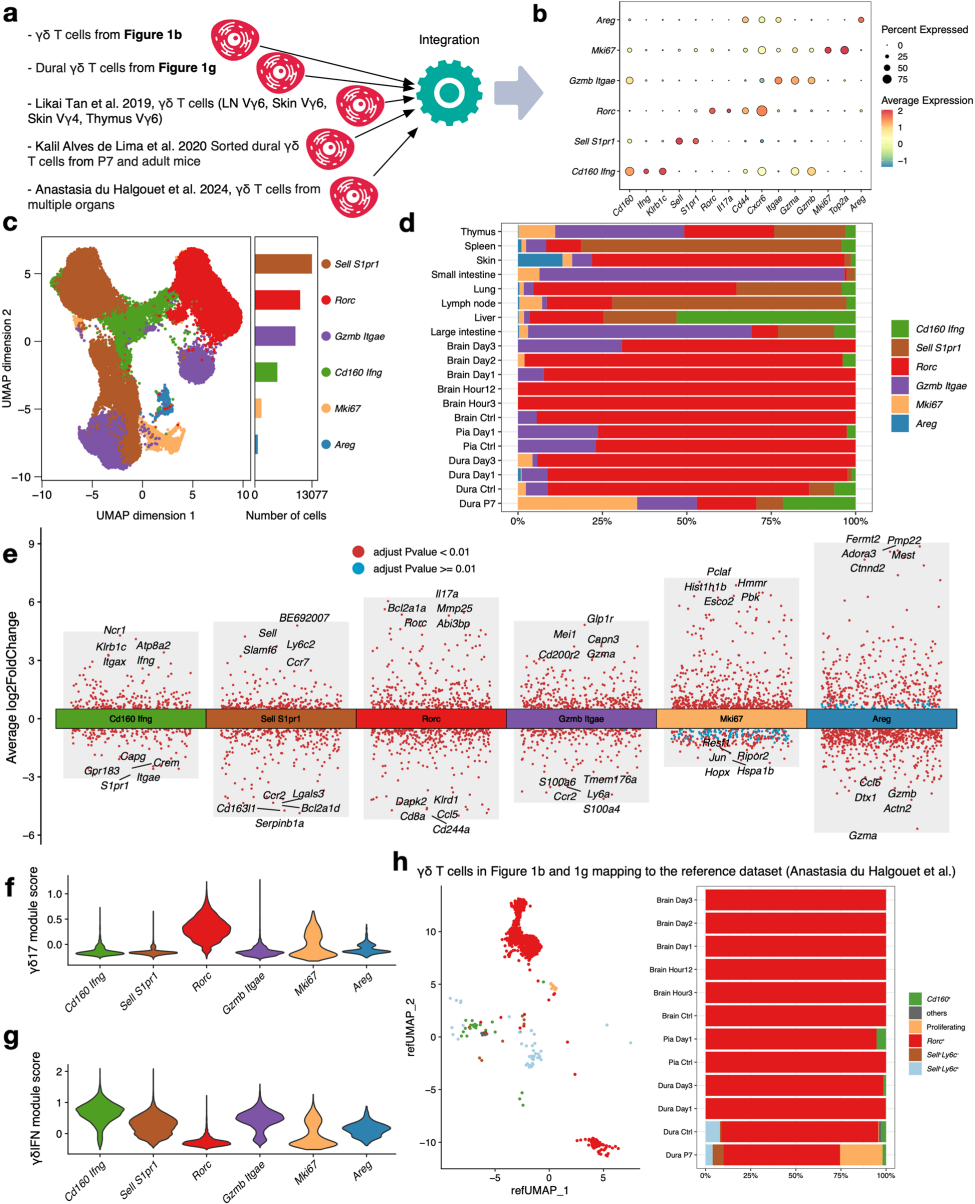
Integrated analysis identifies a γδ17-phenotype shared by meningeal and brain-infiltrating γδ T cells post-stroke. **a**. Workflow for integrating γδ T cells from multiple scRNA-seq datasets. **b**. Dot plot of canonical marker genes used for phenotype annotation. Dot color indicates scaled average expression, and dot size represents the percentage of cells expressing the gene. **c**. Uniform Manifold Approximation and Projection (UMAP) visualization of the integrated γδ T cell dataset, colored by subcluster identity. The bar plot shows the cell count per subcluster. **d**. Distribution and temporal dynamics of γδ T cell subclusters across different tissues and time points in the integrated dataset. **e**. Volcano plots highlighting the top five cluster-specific marker genes for each γδ T cell subcluster. **f**-**g**. Violin plot depicting the distribution of the γδ17 and γδIFN gene module score across different subclusters. **h**. Phenotypic annotation of brain and meningeal γδ T cells via reference mapping to an annotated dataset from du Halgouet et al. Abbreviations: LN, lymph node; UMAP, Uniform Manifold Approximation and Projection

To investigate the transcriptional heterogeneity and niche-specific features of γδ T cells in the brain and meninges during homeostasis and stroke, we integrated them with multiple large-scale γδ T cell datasets (Fig. 3a). After integration and clustering, clusters were annotated based on the expressions of key markers displayed in Fig. 3b. These annotations were modified based on the classification criteria raised by du Halgouet et al. [22]. A total of 6 subclusters of γδ T cells were annotated (Fig. 3c), and the distribution of γδ T cell subclusters varied substantially across organs. Notably, the *Rorc* cluster constituted the predominant subset in the brain, dura, and pia mater in homeostasis and post-stroke, except for dura from P7 mice (Fig. 3d). The top-5 marker genes for each cluster are shown in Fig. 3e. As shown in Figs. 3f and g, the *Rorc* cluster had the highest γδ17 and lowest γδIFN module scores compared with other clusters, indicative of an IL-17-producing phenotype. The accuracy of annotations was further confirmed by the reference mapping method [22], which yielded consistent results (Fig. 3h).

**Fig. 4:**
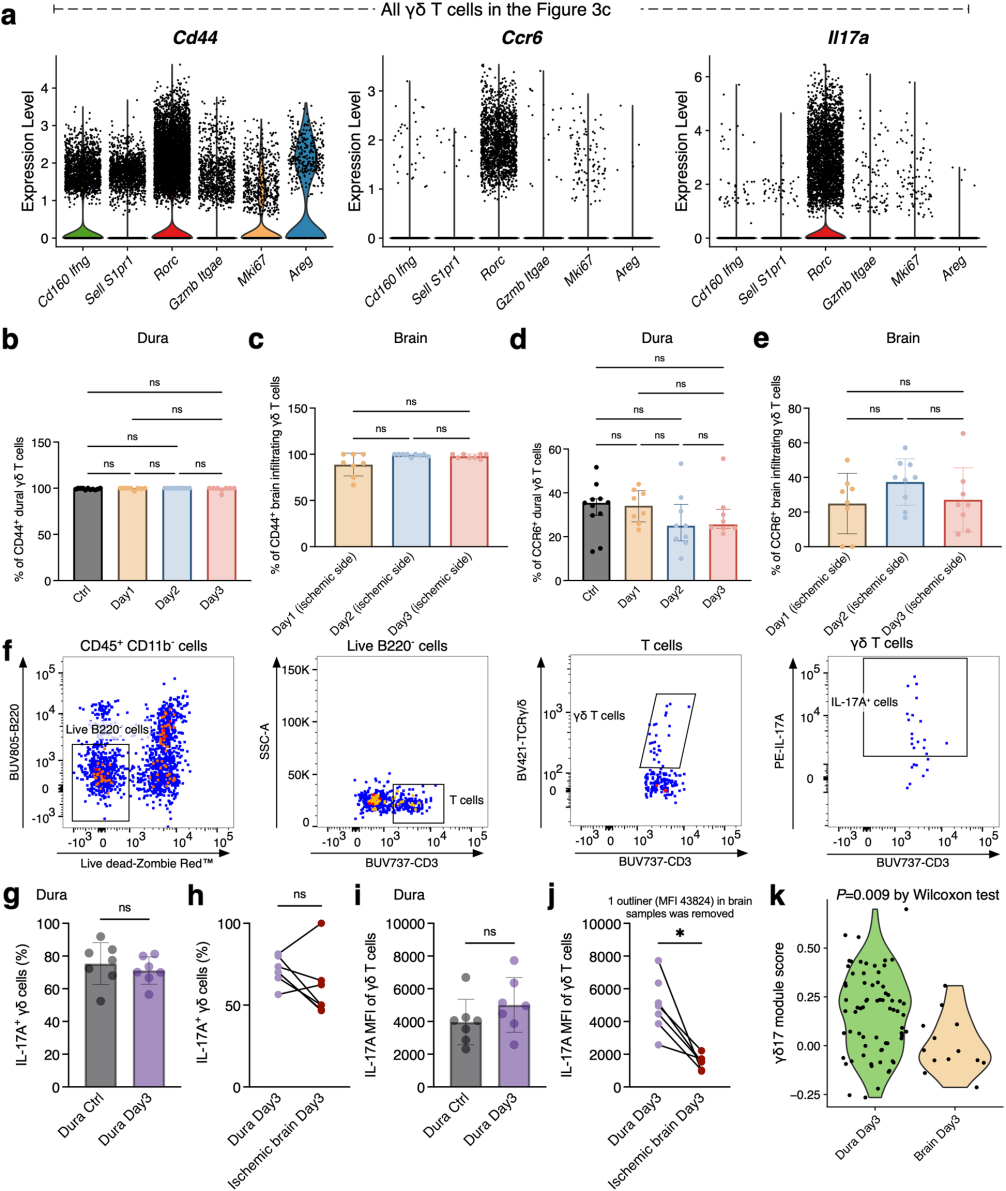
Comparisons on phenotypes between dural and brain-infiltrating γδ T cells post-stroke. **a**. Violin plots showing scaled expression of *Cd44*, *Ccr6*, and *Il17a* in the integrated γδ T cell dataset. **b**-**e**. Proportions of CD44^+^ and CCR6^+^ γδ T cells in the dura and ischemic brain were similar at baseline and across post-stroke time points. Frequencies of CD44⁺ γδ T cells in the dura (**b**) and brain (**c**). Percentages of CCR6⁺ γδ T cells in the dura (**d**) and brain (**e**). For **b**-**e**, data are mean ± SD; *n* = 8–11 *Tcrδ*‑GDL mice per group across 3–4 independent experiments. Significance was determined by ANOVA or Kruskal-Wallis test with appropriate multiple comparisons, based on normality and variance homogeneity. **f**. Gating strategy for detecting IL‑17A⁺ γδ T cells after stimulation. **g**. Proportion of IL‑17A⁺ dural γδ T cells was similar between the control and the day 3 group. **h**.​ Proportion of IL‑17A⁺ γδ T cells did not differ between the dura and the matched ischemic brain at day 3. **i**.​ Median fluorescence intensity (MFI) of IL‑17A in dural γδ T cells was comparable between the control and the day 3 group. **j**.​ IL‑17A MFI values were significantly lower in brain-infiltrating γδ T cells than those from the matched dura at day 3 (one outlier in the brain was excluded, MFI = 43,824). For g-j, data are mean ± SD; *n* = 6–7 *Tcrδ*‑GDL mice per group across 3 independent experiments. Statistical analysis used unpaired t‑test, Wilcoxon matched-pairs signed rank test, or paired t‑test as appropriate. **k**. Comparison of γδ17 module scores between the dural- and brain-infiltrating- γδ T cells at day 3 post-stroke. The Wilcoxon test was used to check the significance of the difference. Abbreviations: ANOVA, analysis of variance; SD, standard deviation; ns, non-significant; IL, Interleukin; MFI, median fluorescence intensity

Flow cytometric experiments were arranged to validate the high expression of *Cd44*, *Ccr6*, and *Il17a* observed in the *Rorc* cluster (Fig. 4a), and we found that substantial proportions of γδ T cells in the dura and brain, regardless of homeostatic or post-stroke state, were positive for CD44 and CCR6 (Fig. 4b-e). No significant differences in these positive rates were detected across time points. Besides, the *Cd163l1* (*Scart1*) gene was highly expressed in γδ T cells from the brain and meninges (Supplemental Fig. 3), which has been established to be a key marker of Vγ6^+^cells [21]. Only a few cells were positive for the *Scart2* gene (marker for Vγ4^+^cells). Flow cytometric analysis of stimulated γδ T cells (Fig. 4f) revealed that a median of ~ 70% (dura) and 50% (brain) were IL-17A⁺ at day 3 (Figs. 4g-h). While the median fluorescence intensity of IL-17A in γδ T cells remained comparable in the dura between baseline and day 3 (Fig. 4i), it was significantly reduced in γδ T cells isolated from the ischemic brain at day 3 compared with γδ T cells from the corresponding dura (Fig. 4j). This indicates a functional disparity in cytokine production between dural and brain-infiltrating γδ T cells, despite their shared major phenotype. A significant difference in γδ17 gene signature module scores between dura and brain at 3 days post-stroke was also observed (Fig. 4k).

In summary, integrated scRNA-seq and flow cytometry analyses revealed that the majority of both brain-infiltrating and meningeal cells before and after stroke exhibited an activated Vγ6^+^ γδ17 phenotype. However, a functional disparity in cytokine production existed between the two compartments at day 3.

### Spatiotemporal dynamics of tissue residency and migratory capacities in dural and brain-infiltrating γδ T cells following stroke

**Fig. 5:**
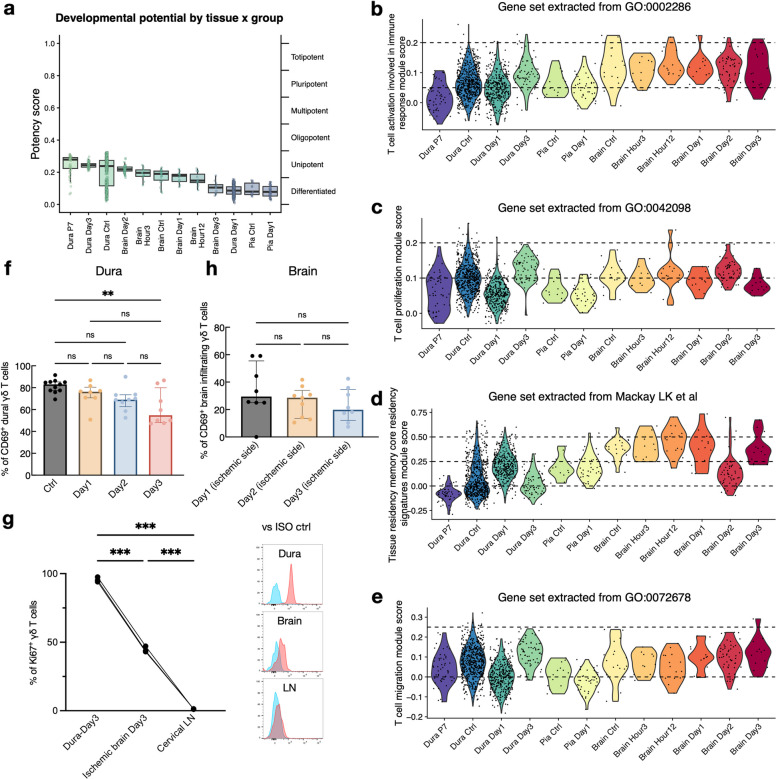
Stroke-induced phenotype changes in activation, proliferation, tissue residency, and migration of dural and brain-infiltrating γδ T cells. **a**. Developmental potential of brain- and border-associated γδ T cells predicted by CytoTRACE2, visualized by tissue/experimental group. **b**. Module score analysis of the Gene Ontology (GO) term “T cell activation involved in immune response” (GO:0002286) across γδ T cell subclusters. **c**. Module score analysis of the GO term “T cell proliferation” (GO:0042098) across γδ T cell subclusters. **d**. Module score analysis of the Tissue residency memory core residency gene signatures (extracted from Mackay LK et al.) across γδ T cell subclusters. **e**.​ Display of the module scores for the “T cell migration” term (GO:0072678) across γδ T cell subclusters. **f**.​ Frequency of CD69⁺ dural γδ T cells was significantly lower at day 3 compared with the control group. **g**. Frequency of Ki-67⁺ γδ T cell was significantly higher in the dura than in the ischemic brain and cervical lymph nodes at day 3 post-stroke. Data are mean ± SD; *n* = 3 C57BL/6 mice from one experiment. Significance was tested by the repeated measures analysis of variance (ANOVA). Representative histograms of Ki-67 staining (red) versus isotype control (blue) in the indicated tissues. **h**. Frequency of CD69⁺ brain-infiltrating γδ T cells remained similar across baseline and post-stroke time points. For f and h. Data are median ± IQR; *n* = 8–11 *Tcrδ*‑GDL mice per group across 3–4 independent experiments. Significance was determined by the ANOVA test with appropriate multiple comparisons. Abbreviations: SD, standard deviation; GO, Gene Ontology; ISO, isotype control; LN, lymph node; ANOVA, analysis of variance; ns, non-significant

We performed an analysis on the developmental potential of γδ T cells across the brain and border regions with the CytoTRACE2 R package. The results revealed that P7 dura exhibited the highest potency (Fig. 5a). Interestingly, the brain-infiltrating γδ T cells at day 3 post-stroke were in a more differentiated state, as reflected by their lower developmental potential relative to dural γδ T cells at the same time point.

Enrichment analysis was performed on region- and time-specific upregulated genes, and the enriched pathways are presented in Supplemental Fig. 4. We also performed a comprehensive module‑score analysis to characterize differences in the immunophenotype of γδ T cells in the brain and border‑associated regions, including T cell activation (Fig. 5b), proliferation (Fig. 5c), tissue residency (Fig. 5d), and migration (Fig. 5e). The analysis showed that dural γδ T cells exhibited reduced activation, proliferation, and migration capacities at day 1, followed by an increase at day 3 relative to baseline and day 1, reflecting a dynamic impact of stroke on these cells. In parallel, the tissue‑residency module scores of dural γδ T cells were elevated on day 1 and declined on day 3, indicating enhanced migratory ability on day 3, which was further supported by flow‑cytometric analysis of the tissue‑retention marker CD69 [48], as shown in Fig. 5f.

In contrast, brain‑infiltrating γδ T cells displayed consistently higher T‑cell activation (Fig. 5b), relatively lower proliferation module scores by day 3 (Fig. 5c), decreased tissue‑residency capacity on day 2 (Fig. 5d), and stably elevated migration module scores throughout stroke progression (Fig. 5e). To validate these observations, we assessed Ki‑67 staining in γδ T cells on day 3 post‑stroke by flow cytometry (Fig. 5g). The relatively low proliferative rate in the brain suggests that local self‑renewal is unlikely to fully explain the rapid accumulation of γδ T cells. Furthermore, the lower percentage of CD69^+^ γδ T cells in the brain compared with the dura also points to a migration‑related origin rather than local proliferation (Fig. 5h).

Collectively, these findings imply that brain‑infiltrating γδ T cells likely derive from sources other than local proliferation, whereas dural γδ T cells undergo a phenotypic shift toward increased mobility and reduced tissue retention during stroke progression.

### Interactions between dural γδ T cells and CD45-negative cells might be enhanced on day 3 post-experimental stroke

**Fig. 6:**
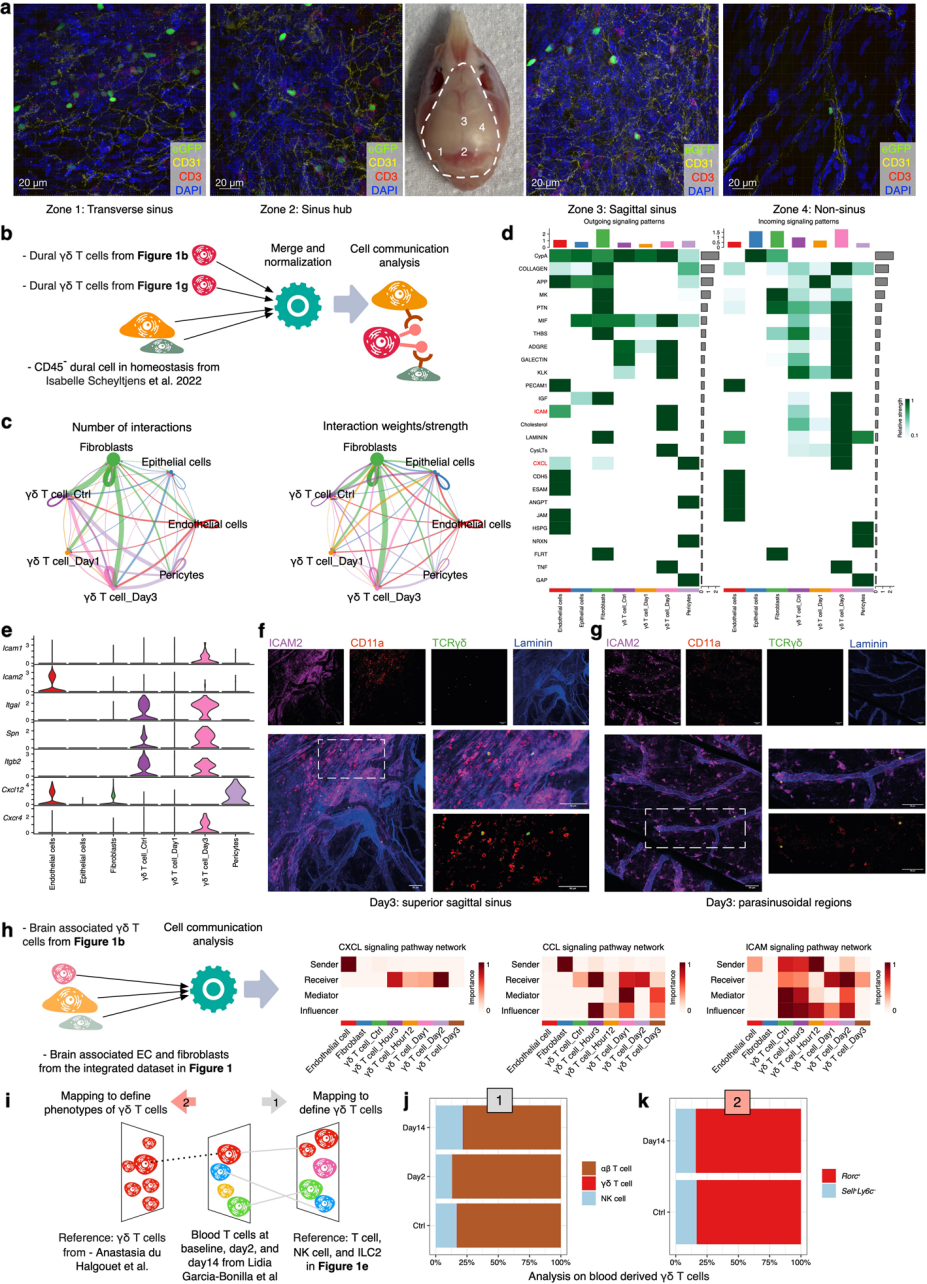
Enhanced interactions between dural γδ T cells and CD45-negative cells on day 3 might exist. **a**. Representative confocal micrographs of γδ T cells (green) in different regions of the dural meninges. Nuclei are counterstained with DAPI (blue). CD31 (yellow); CD3 (red); Scale bar, 20 μm. **b**. Workflow for cell–cell communication analysis between γδ T cells at different time points after stroke and CD45⁻ cells in the dura. **c**. Total number and interaction strength of predicted cell–cell communications, analyzed by the CellChat R package across cell types and post-stroke time points. **d**. Heatmap showing outgoing and incoming signaling patterns across cell populations. **e**. Violin plots depicting key genes in the intercellular cell adhesion molecule (ICAM) and C-X-C motifChemokine ligand (CXCL) pathways. **f**-**g**. Representative confocal micrographs of γδ T cells and endothelial cells at day 3 post-stroke in the superior sagittal sinus (**f**) and adjacent parasinusoidal region (**g**) of the dural meninges. ICAM2 (purple); CD11a (red); TCRγ/δ (nuclear eGFP signal in the *Tcrδ-*H2BEGFP mice, green); Laminin (blue); Scale bar, 50 μm. **h**. Exploration on the interactions between brain-infiltrating γδ T cells and brain-derived endothelial cells and fibroblasts. **i**-**k**. Identification and phenotypic annotation of blood-associated γδ T cells via the reference mapping method. Abbreviations: eGFP, enhanced green fluorescence protein; EC, endothelial cell; ICAM, intercellular cell adhesion molecule; CXCL, C-X-C motif Chemokine ligand; CCL, C–C Motif Chemokine ligand; CXCR, C-X-C motif Chemokine Receptor; NK, natural killer; ILC, innate lymphoid cell

To explore potential mechanisms underlying dural γδ T cell recruitment, we examined their spatial distributions. Immunofluorescence analysis revealed that γδ T cells were enriched near CD31⁺ vascular structures, particularly in the venous sinus region of the dura (Fig. 6a), suggesting potential interactions with endothelial cells and/or pericytes. To decipher potential receptor-ligand pairs involved in the recruitment of γδ T cells, we carried out cell communication analysis between dural CD45^−^ cells and γδ T cells (Fig. 6b). The results indicated that dural γδ T cells at day 3 post-stroke showed stronger interactions with other CD45^−^ cells (Fig. 6c). Further analysis of signaling pathways revealed enhanced activity of the Intercellular Cell Adhesion Molecule (ICAM) and C-X-C motif Chemokine Ligand (CXCL) pathways in dural γδ T cells at day 3 post-stroke (Fig. 6d, Supplemental Fig. 5), both of which are implicated in immune cell migration. Key genes involved in these two pathways are shown in Fig. 6e, including *Icam2* and *Cxcl12* expressed by endothelial cells, along with their corresponding receptors, *Itgal* and *Cxcr4*, respectively, in dural γδ T cells.

To validate the interactions between dural γδ T cells and endothelial cells, we performed additional immunofluorescence experiments on whole-mount dura mater from *Tcrδ*-H2BEGFP mice three days after tMCAO. Cell–cell communication analysis using CellChat identified *Cd11a* expressed by γδ T cells and *Icam2* expressed by endothelial cells as a potential ligand-receptor pair. These candidates were therefore selected for validation by immunofluorescence staining. CD11a⁺ dural γδ T cells were observed in proximity to ICAM2⁺ vessels within the superior sagittal sinus (Fig. 6f) and adjacent parasinusoidal regions (Fig. 6g), suggesting potential interactions between γδ T cells and endothelial cells post stroke. We also performed flow cytometric experiments to compare the percentages of CD11a (ITGAL) and CXCR4 on dural γδ T cells between baseline and day 3 groups. However, the differences between the two groups did not reach statistical significance, although with an increasing trend in the day 3 group (Supplemental Fig. 6).

We further examined interactions between brain-infiltrating γδ T cells and brain-associated endothelial cells/pericytes to explore possible infiltration routes. The analysis revealed that signaling pathways related to immune‑cell migration—including CXCL, CCL (C–C Motif Chemokine Ligand), and ICAM—were dynamically regulated during stroke progression (Fig. 6h). These results suggest that γδ T cells may initiate brain infiltration as early as 3 h post‑stroke through interactions with endothelial cells and/or pericytes.

The circulating blood compartment is a potential source of brain-infiltrating γδ T cells. We therefore included γδ T cells from the circulating blood into our analysis. To identify γδ T cells from blood scRNA‑seq data, we employed a reference‑mapping strategy (Fig. 6i). Following annotation, we identified 31 blood‑derived γδ T cells from a total of 8,470 T cells and NK cells (Fig. 6j). These blood‑derived γδ T cells were then mapped to a large‑scale γδ T‑cell reference, which showed that the predominant phenotype remained *Rorc*⁺ (Fig. 6k). These results indicated that blood‑derived γδ T cells might be a potential source for the brain-infiltrating γδ T cells. However, the interpretation had to be cautious, considering the low percentage of γδ T cells identified in the blood scRNAseq data.

In summary, the findings presented in this section point to potential future research directions concerning the migration of γδ T cells within the dura and their infiltration into the brain.

## Discussion

In this study, we conducted a comprehensive analysis of γδ T cells in the brain and its border regions during the acute phase of experimental stroke. By analyzing publicly available datasets alongside unpublished in-house data, we observed that the majority of γδ T cells residing in the meninges and infiltrating the central nervous system, both under homeostatic conditions and following stroke, were *Rorc*⁺ and belonged to the Vγ6⁺ γδ17 cell subset.

Besides, we notice that meningeal γδ T cells undergo local activation and proliferation within the dura following experimental stroke. Dural γδ T cells exhibited higher expression of Ki-67 compared with brain-infiltrating γδ T cells at day 3 post-experimental stroke, together with high developmental potential. A comparable response has been described in sterile inflammatory models of the skin, where γδ T cells have been shown to proliferate under inflammatory conditions in an IL-1β- and IL-23-dependent manner [49]. Interestingly, the activation of migratory pathways was accompanied by a loss of tissue-residency markers such as CD69 [50]. Consistent with this observation, flow cytometric analyses revealed a significant decrease in dural γδ T cell numbers beginning on day 2 after stroke, which reminded us that these γδ T cells may be recruited out of the meninges [51]. Moreover, immunofluorescence experiments showed that numerous dural γδ T cells resided adjacent to CD31^+^ vessels, suggesting possible interactions between γδ T cells and endothelial cells/pericytes. Signaling pathway analyses of transcriptomic data demonstrated enhanced interactions between dural γδ T cells and endothelial cells/fibroblasts via ICAM- and CXCL-dependent pathways following ischemia. Additional immunofluorescence experiments confirmed the expressions of ICAM2 and ITGAL on dural endothelial cells and dural γδ T cells at day 3 post-stroke, respectively. To sum up, our findings revealed that dural γδ T cells might be activated and leave the dura via interacting with endothelial cells during stroke progression.

While we observed a concomitant decrease in meningeal γδ T cells alongside an accumulation of γδ T cells in the ischemic brain, we cannot conclude that dural γδ T cells represent the primary source of brain-infiltrating γδ T cells after stroke, as Vγ6⁺ γδ17 T cells are also present in peripheral lymph nodes, the spleen, small intestine, and skin [21, 52]. A previous study has shown that IL-17⁺ γδ T cells can migrate from the intestine to the meninges and influence ischemic injury after stroke [53]. Besides, our previously published work indicated that γδ T cell infiltration into the brain parenchyma began within the first 24 h after experimental stroke, with a significant increase detected on day 2 after stroke modeling [5, 16]. Based on findings from the current study, the interaction between brain-infiltrating γδ T cells and brain-associated endothelial cells/fibroblasts was upregulated as early as 3 h post-stroke. This process occurred before the upregulation of migratory pathways and the numerical reduction in the dura. This temporal dissociation suggests that γδ T cells infiltrating the ischemic brain before day 1 are likely derived from peripheral sources rather than the dura, such as the bloodstream. Supporting the notion that brain-infiltrating γδ T cells can be recruited from the blood, we found *Rorc*⁺ γδ T cells in the peripheral blood under homeostasis and post-stroke.

One possible explanation for the aforementioned results might be that γδ T cells infiltrate the brain in multiple waves. The initial wave is likely not of dural origin. Subsequent waves could potentially egress from the dura, enter the bloodstream, and then infiltrate the ischemic parenchyma. Given these uncertainties, future studies using refined imaging techniques and specialized animal models, including photoconversion-based tracking, will be necessary to clarify the precise migratory routes and tissue origins of γδ T cells following stroke.

Another central question that cannot be addressed with our current methodology is whether γδ T cells can migrate directly from the dura mater into the brain parenchyma. One potential route could involve recently described arachnoid cuff exit (ACE) points. In a mouse model of multiple sclerosis, immune cell migration from the dura mater into the subarachnoid space via ACE points has recently been proposed [11]. However, this concept remains controversial, as other studies question whether immune cells can cross the arachnoid barrier from the dura into the brain parenchyma [54]. Alternatively, dural γδ T cells may migrate to the cervical lymph nodes, as described for dermal IL-17A-producing γδ T cells [55]. Future studies using in vivo labeling of dural immune cells in mouse models with photoconvertible reporters, combined with two-photon imaging through cortical windows, may help clarify the migratory fate of dural γδ T cells.

A closer immunophenotypic analysis also revealed that brain-infiltrating γδ T cells differed from meningeal γδ T cells by expressing lower levels of IL-17A as measured by mean fluorescence intensity. Whether IL-17A produced in the meninges exerts a comparable pro-inflammatory effect in the context of stroke remains unclear. We and others have previously shown that hyperacute IL-17A production after stroke is detrimental due to the local excessive amplification of neutrophil infiltration into the ischemic brain parenchyma [5, 6, 16]. This synergistic stimulation is particularly relevant in IL-17 receptor-expressing brain-resident cells such as astrocytes, where IL-17A amplifies chemokine production. Given that IL-17A-mediated effects during hyperacute sterile inflammation depend on synergy with IL-1β and TNF-α, both of which are primarily produced by microglia within the brain parenchyma [56, 57], it is plausible that parenchymal IL-17A plays a more prominent role than meningeal IL-17A in driving the initial inflammatory response in ischemic brain tissue.

From a physiological perspective, basal IL-17A production in the meninges remains important. Previous studies have suggested that IL-17A modulates anxiety-related behavior and memory under homeostatic conditions in mice [14, 15]. Against this background, stroke-induced alterations in the meningeal immune compartment may be particularly relevant for recovery and regeneration and should therefore be investigated in studies extending beyond the hyperacute phase to examine changes in dural γδ T cells during post-stroke recovery.

In summary, this study integrated multiple publicly available scRNA-seq datasets and validated key findings using flow cytometry and immunofluorescence. The accuracy of γδ T cell subtype annotation was supported by both reference mapping and data integration approaches. Collectively, these results provide a foundation for further investigation of the meningeal immune compartment in hyperacute stroke.

However, several limitations should be acknowledged. Direct evidence of cellular migration remains lacking. In addition, the relatively low abundance of γδ T cells in the scRNA-seq datasets may introduce bias into the analysis. Moreover, the findings are primarily based on transcriptomic data, and the study is restricted to the acute phase (0–3 days), without assessment of γδ T cell dynamics during the recovery phase. Future studies incorporating direct cellular tracing at the meningeal-brain interface, such as in vivo two-photon imaging, will be necessary to further validate these observations. Furthermore, potential contamination with arachnoid mater tissue during dura isolation cannot be excluded, although the procedures were performed according to established methods reported in the literature [9, 23, 44].

## Conclusions

This study provides a comprehensive analysis of the spatiotemporal dynamics and functional reprogramming of γδ T cells in the meninges and brain during the early phase of experimental stroke. Collectively, our data suggest that stroke-associated sterile inflammation differentially programs γδ T cell reactivities depending on their anatomical compartment. Given the pleiotropic roles of meningeal γδ T cells in both homeostasis and inflammation, further studies are required to determine whether the effects of meningeal γδ T cells are confined to the meningeal immune compartment or whether they also contribute to excessive post-ischemic inflammation within the brain parenchyma.

## Supplementary Information

Supplementary Material 1.

Supplementary Material 2.

Supplementary Material 3.

Supplementary Material 4.

## Data Availability

The codes generated for this study are shown in the Additional file 1. All information on the mice used in this study is shown in Additional file 2. The processed flow cytometric results are attached to the Additional file 3. All publicly available single-cell RNA sequencing datasets analyzed in this study were retrieved from the Gene Expression Omnibus database as provided by the original studies, which were also mentioned in the Methods section. The integrated scRNAseq data and in-house γδ T cells used in the current study are available from the Google Drive (Link: https://drive.google.com/drive/folders/1G5p2NjEPxWSxETw0hckLma45-9xYKylg?usp=sharing). The dataset supporting the conclusions of this article is included within the article and its additional files.

## Abbreviations

IL: Interleukin
scRNAseq: Single-cell RNA sequencing
tMCAO: Transient middle cerebral artery occlusion
GEO: Gene Expression Omnibus
NK: Natural killer
ILC: Innate lymphoid cells
UMAP: Uniform Manifold Approximation and Projection
eGFP: Enhanced green fluorescence protein
MRI: Magnetic resonance imaging
PBS: Phosphate-buffered saline
DMEM: Dulbecco's Modified Eagle Medium
CD: Cluster of Differentiation
TCR: T cell receptor
IFN: Interferon
CCR: C–C Motif Chemokine Receptor
CXCR: C-X-C motif Chemokine Receptor
GEM: Gel beads-in-emulsion
GEX: Gene expression
CSP: Cell surface protein
ANOVA: Analysis of variance
ICAM: Intercellular cell adhesion molecule
CXCL: C-X-C motif Chemokine Ligand
CCL: C–C Motif Chemokine Ligand
ITGAL: Integrin alpha L
ACE: Arachnoid cuff exit
