# Supplementary material for: Single-cell omics and flow cytometry identify distinct immune states of dural and brain-infiltrating IL-17-producing γδ T cells after experimental stroke
Source: J Neuroinflammation. 2026 Apr 9;23:127. doi: 10.1186/s12974-026-03794-3 (PMC13088706; doi:10.1186/s12974-026-03794-3)
Supplement: Supplementary file 4 — Supplementary Material 4. [file 12974_2026_3794_MOESM4_ESM.pdf]

Single-cell omics and flow cytometry identify distinct immune states of dural and brain-infiltrating IL-17-producing γδ T cells after experimental stroke

Mingming Zha, MD[1,2*], Alina Jander, MD[1*], Haodi Cai, MD[1,3], Marius Piepke, MD[1], Karoline Degenhardt, PhD[1], Leo Winter, MDc[1], Tim Magnus, MD[1], Mathias Gelderblom, MD[1#]

[1] Department of Neurology, University Medical Center Hamburg-Eppendorf, Hamburg, Germany

[2] Department of Neurology, First Affiliated Hospital, School of Medicine, Zhejiang University, Zhejiang, China

[3] Department of Neurology, the First Affiliated Hospital of Soochow University, Jiangsu, China

[*]Contributed equally

[#]Corresponding author: PD Dr. Mathias Gelderblom

Department of Neurology

University Medical Center Hamburg-Eppendorf

Martinistrasse 52, 20246 Hamburg, Germany

E-mail: m.gelderblom@uke.de

Supplemental Figure 1. Process of the dataset in the **Figure 1**

First round

Data from five studies
Clustering & annotation

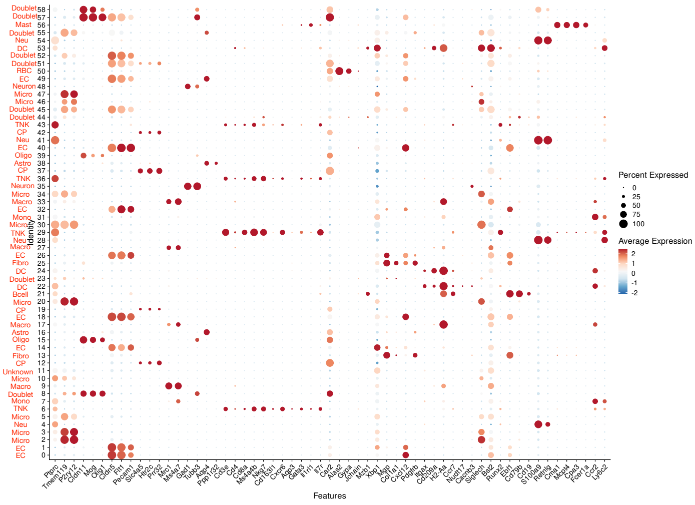

Exclude non-immune and low quality cells

Extract brain associated endothelial cells and fibroblasts for downstream cell communication analysis

Second round

Clustering & annotation

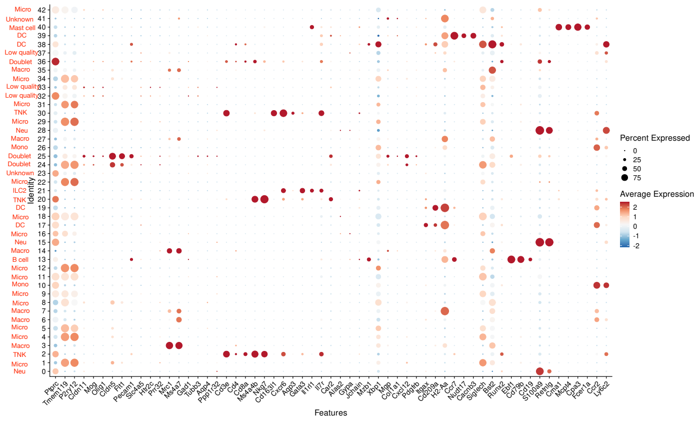

Exclude doublet, unknown, and low quality cells

Third round

Clustering & annotation

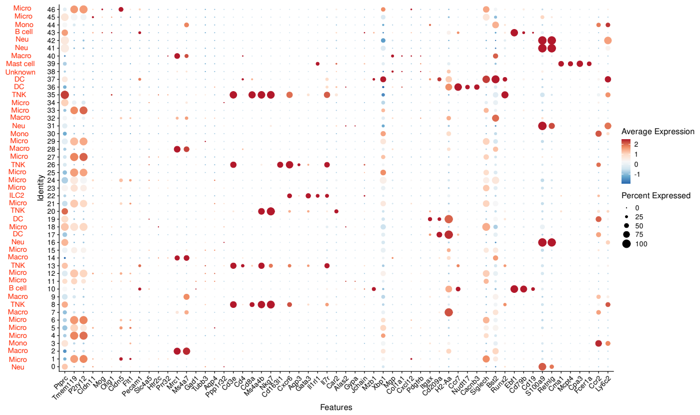

Exclude unknown cluster 38

Final round

Clustering & annotation

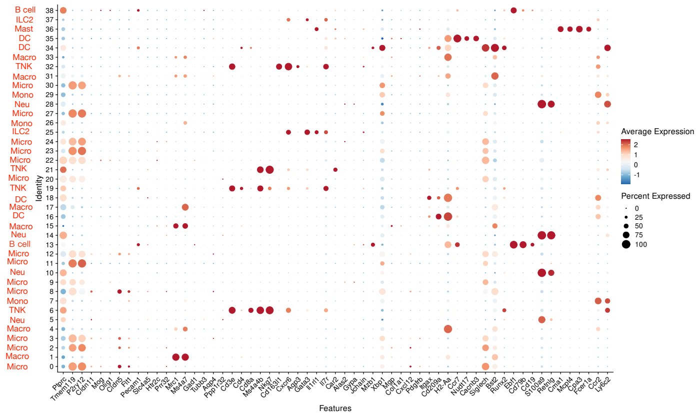

Supplemental Figure 2. Re-clustering and finer annotation of T cells, NK cells, and ILC2 in the **Figure 1e**

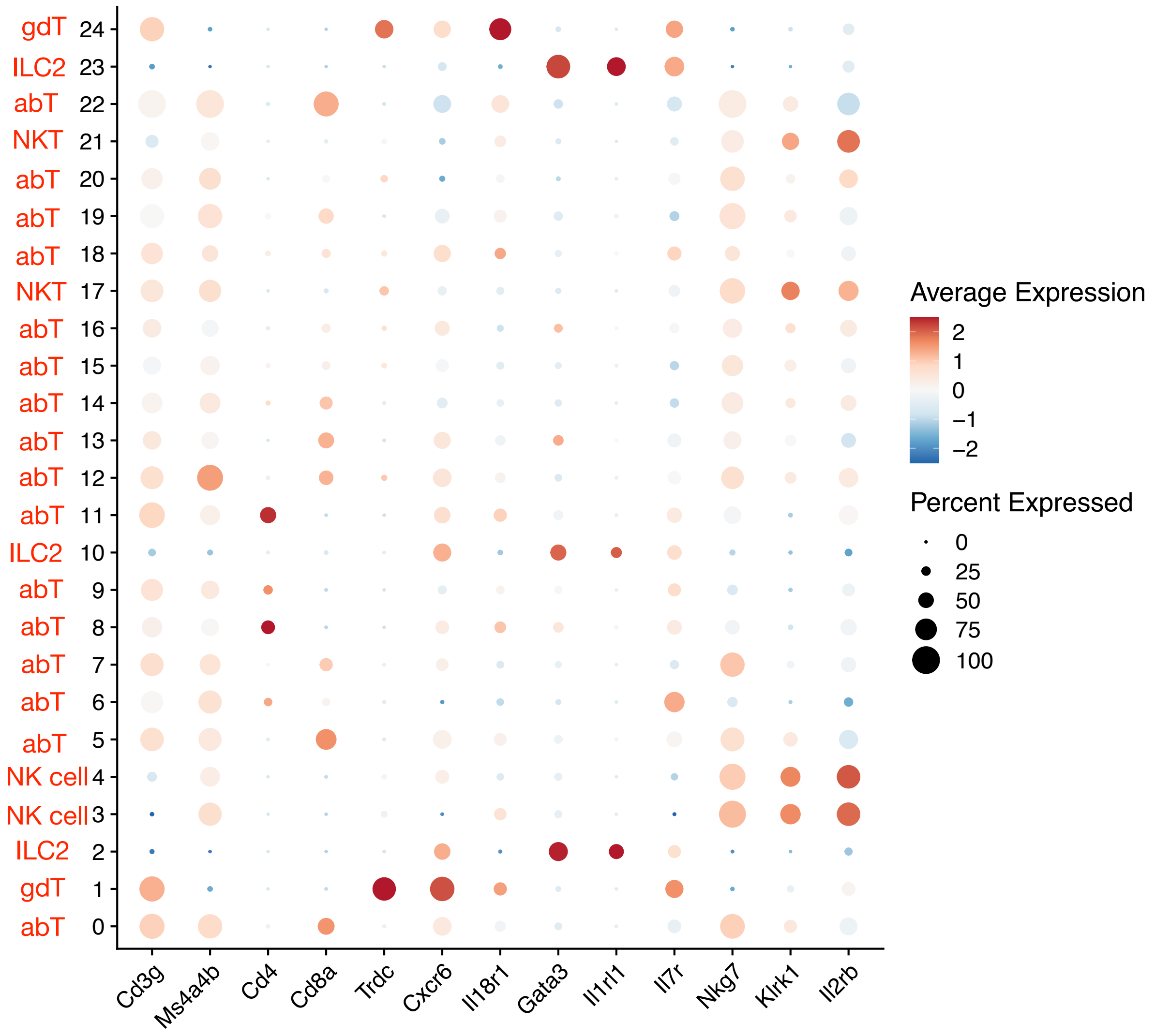

Abbreviation: ILC, innate lymphoid cell.

Supplemental Figure 3. Expression profiles of *Scart1* and *Scart2* genes in γδ T cells from brain and border regions.

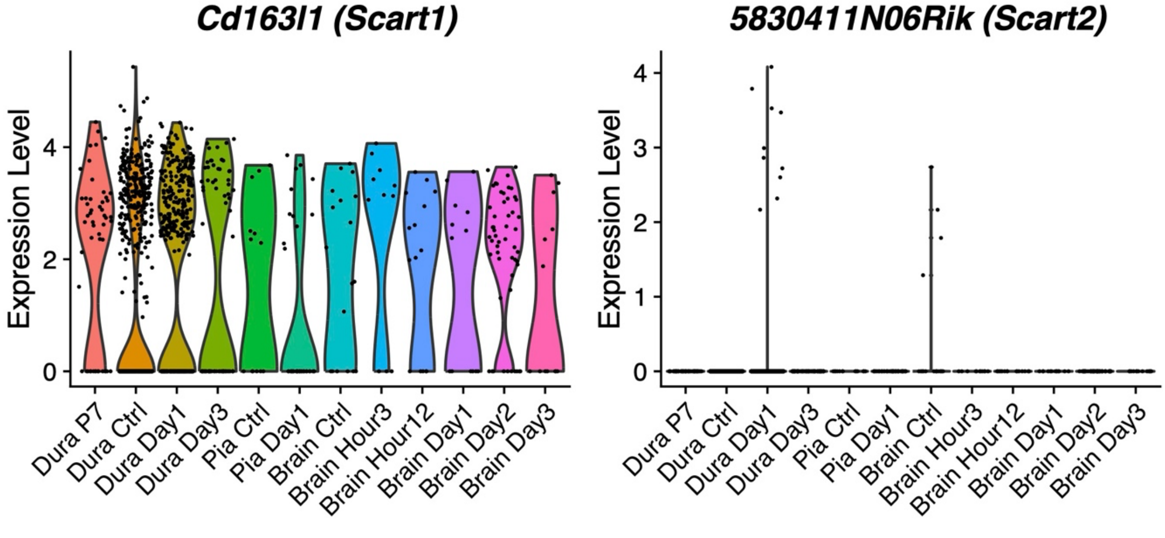

Supplemental Figure 4. Bar plots of the top 10 enriched gene ontology biological process clusters, based on the top 100 differentially upregulated genes for each subtype of γδ T cells

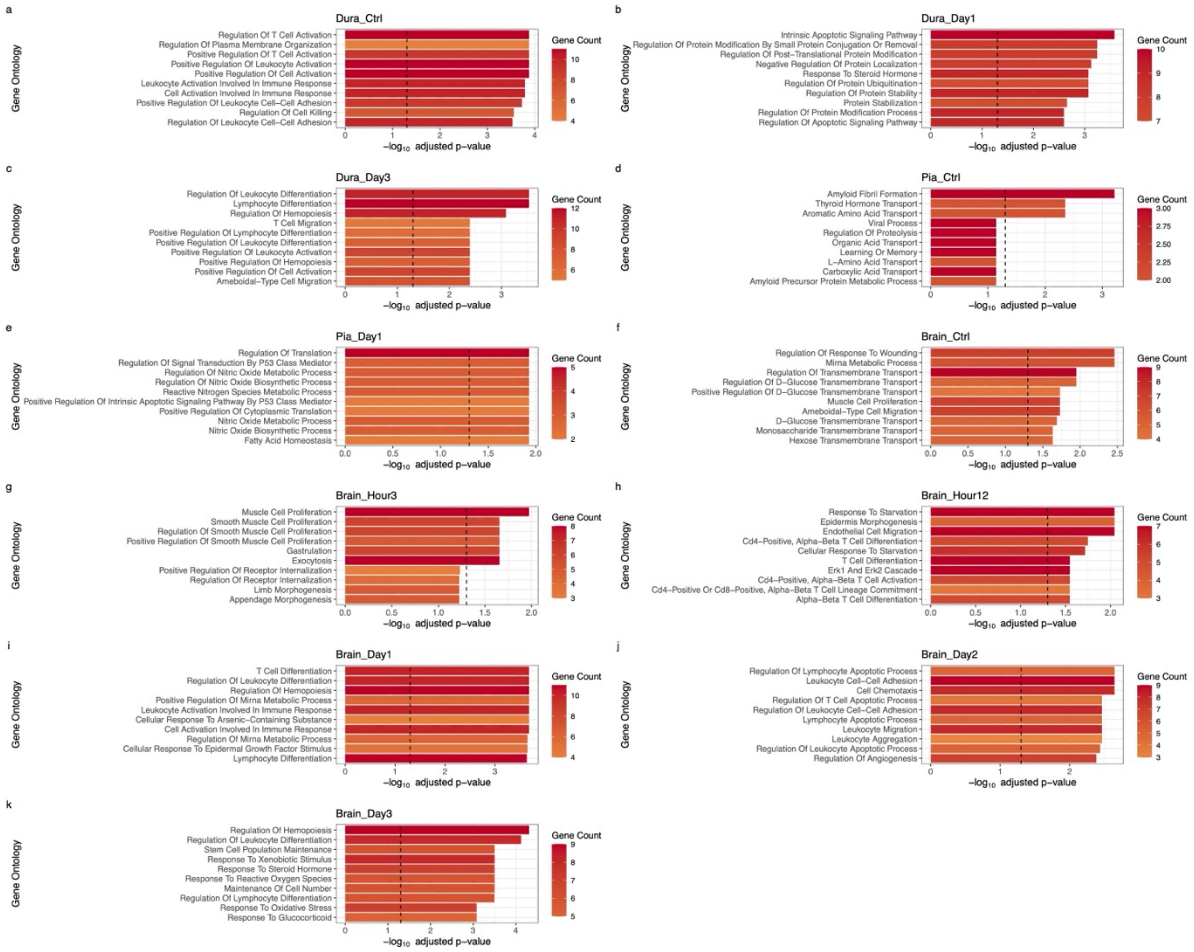

Supplemental Figure 5. Chord diagrams of the ICAM and CXCL pathways among different cell types in the dura

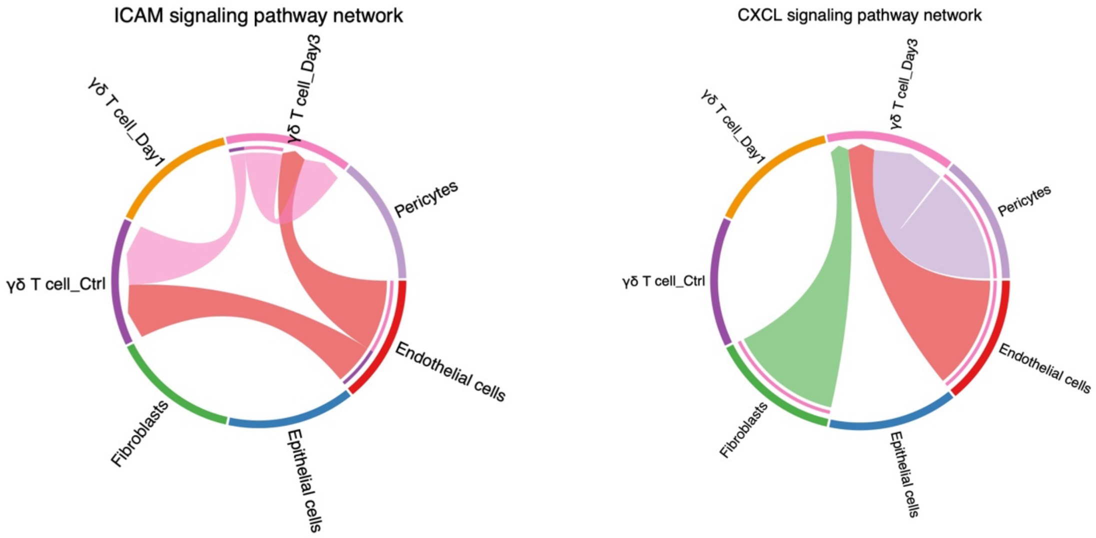

Chord diagrams summarizing ligand-receptor interactions for the intercellular cell adhesion molecule (ICAM) and C-X-C motif chemokine ligand (CXCL) signaling pathways.

Supplemental Figure 6. Percentages of CD11a^{+} and CXCR4^{+} in dura and brain infiltrating γδ T cells at day 3 post-experimental stroke

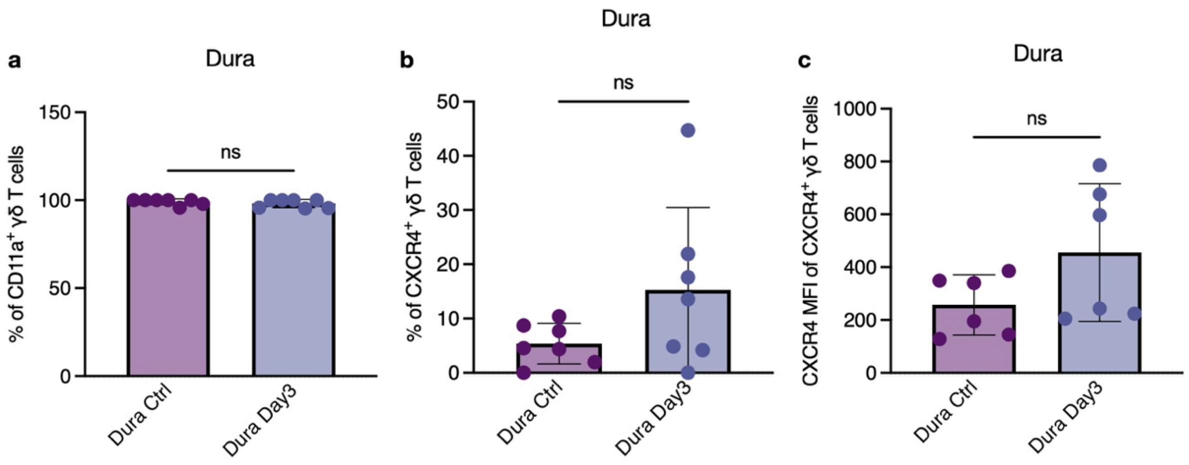

a. Frequencies of CD11a^{+} dural γδ T cells at the baseline versus the day 3 group.

b. Percentages of CXCR4^{+} dural γδ T cells at the baseline versus the day 3 group.

c. CXCR4 median fluorescence intensity (MFI) on dural CXCR4^{+} γδ T cells at baseline and 3 days after experimental stroke. Data are mean ± standard deviation; n = 6-7 *Tcrδ*-GDL mice per group across 3 independent experiments. Statistics were performed using an unpaired t-test or Mann-Whitney test as appropriate. For panel c, two mice (one from each group) that had no detectable CXCR4^{+} γδ T cells were excluded from the MFI analysis.

Abbreviations: ns, non-significant; MFI, Median fluorescence intensity.

Supplemental Table 1. Immediate early genes derived from published literature.

Fos
Hspa1a
Jun
Fosb
Junb
Egr1
Hspa1b
Ubc
Zfp36
Hspb1
Hsp90aa1
Mt2
Dnajb1
Btg2
Nr4a1
Cebpd
Hspa8
Mt1
Ier2
Dnaja1
Socs3
Atf3
Jund
Cebpb
Id3
Ppp1r15a
Hspe1
Cxcl1
Dusp1
Hsp90ab1
Nfkbia

Hsph1

Origin: Scheyltjens I, Van Hove H, De Vlaminck K, Kancheva D, Bastos J, Vara-Perez M, et al. Single-cell RNA and protein profiling of immune cells from the mouse brain and its border tissues. Nat Protoc. 2022;17(10):2354-88.

Supplemental Table 2. Gene symbols of T cell activation involved in immune response gene ontology term (GO:0002286)

Rab27a
Atp7a
Bcl3
Bcl6
Cd69
Cd81
Ccr2
Ccr7
Eomes
F2rl1
Fcer1g
Fgl2
Brd2
Gata3
H2-M3
H2-DMb1
H2-DMb2
Hlx
Hmgb1
Icam1
Ifna1
Ifna11
Ifna2
Ifna4
Ifna5
Ifna6
Ifna7
Ifna9
Ifnab
Ifnb1

Ifng
Cd74
Il12b
Il18
Il18r1
Il2
Il4
Il4ra
Il6
Il6ra
Irf1
Irf4
Itgal
Jak3
Lef1
Lgals1
Lgals3
Loxl3
Anxa1
Ly9
Smad7
Ascl2
Cd46
Mdk
Men1
Clec4d
Myb
Slc11a1
Pck1
Prkcz
Lcp1
Plxna1

Psen1
Psen2
Ptger4
Rara
Relb
Rora
Rorc
Rps6
Sema4a
Foxp3
Slfn2
Spn
Stat3
Stat4
Stat6
Tgfb1
Trp53
Tnfsf4
Tyrobp
Zfp35
Zbtb7b
Gadd45g
Ccl19
Ceacam1
Eif2ak4
Slamf6
Batf
Apbb1ip
Otud5
Socs5
Clec4e
Mtor

Pf4
Brd4
Tbx21
Il21
Tsc1
Opa1
Stx11
Nfkbiz
Il23a
Trem2
Entpd7
Tmem98
Nckap1l
Foxp1
Havcr2
Ripk2
Kmt2a
Sema6d
Nlrp3
Shb
Ifna13
Ifna16
Ifne
Zc3h12a
Malt1
Tnfsf18
Ifna15
Ifna12
Nfkbid
Il27
Ifnz
Rc3h2

Gpr183
Ep300
Rc3h1
Cracr2a
Ifnk
Ifna14
Gm13271
Gm13283
Gm13272
Gm13276
Gm13277
Gm13275
Mir301
Mir326
Mir873a
H2-Ea

Supplemental Table 3. Gene symbols of T cell proliferation gene ontology term (GO: 0042098)

Abl1
Adk
Ager
Aif1
Arg1
Arg2
Slc7a1
Bax
Bcl6
Bid
Blm
Bmi1
Bmp4
Casp3
Ctnnb1
Ccnd3
Cd151
Ctla4
Cd1d1
Cd1d2
Cd24a
Cd28
Cd37
Cd3e
Cd4
Cd44
Cd59a
Cd6
Cd80
Cd81

Cd86
Cdkn2a
Cebpb
Coro1a
Cxcr4
Ccr2
Ccr7
Cd55
Cd55b
Dlg1
Efnb1
Ephb6
Epo
Erbb2
Fadd
Il4i1
Fkbp1a
Fkbp1b
Fyn
Gja1
Gnrh1
Lilrb4a
Gpam
H2-Aa
H2-DMb1
H2-DMb2
Ptpn6
Hes1
Foxj1
Hmgb1
Ido1
Irgm1

Ifnar2
Ifng
Igf1
Igf2
Igfbp2
Ihh
Il12a
Il12b
Il12rb1
Il15
Il18
Il1a
Il1b
Il2
Il2ra
Il3
Il4
Il6
Il6st
Irf1
Itch
Itgal
Itgam
Itgb2
Jak2
Jak3
Laptm5
Lep
Lgals3
Lgals9
Lipa
Anxa1

Xcl1
Mad1l1
Cd46
Kitl
Msn
Myc
Nck1
Nck2
Cd244a
Slc11a1
P2rx7
Prkcq
Pla2g2d
Pla2g5
Pnp
Ppp3ca
Prkar1a
Mapk8ip1
Prnp
Psmb10
Pten
Ptpn11
Ptpn22
Ptprc
Rac2
Rasgrp1
Rps6
Satb1
Ccl5
Cxcl12
Foxp3
Sftpd

Shh
Slc4a1
Slc4a2
Slfn1
Slfn2
Sos1
Sos2
Spn
Spta1
Stat5a
Stat5b
Syk
Sdc4
Prdx2
Tgfb1
Tgfbr2
Tnfrsf1b
Tnfrsf9
Cd40lg
Cd70
Tnfsf9
Traf6
Tfrc
Trp53
Tsc2
Tnfrsf4
Tnfsf4
Scgb1a1
Vcam1
Wnt4
Zap70
Zbtb7b

Zp3
Ccl19
Tnfsf13b
Ceacam1
Nr5a2
Pla2g2f
Tspan32
Rps3
Slamf1
Sh2d2a
Ebi3
Icosl
Ctps1
Crtam
Tyk2
Elf4
Ripk3
Marchf7
Pdcd1lg2
Zbtb32
Sh3rf1
Ncstn
Il21
Cd274
Ndfip1
Twsg1
Pycard
Peli1
Washc1
Znhit1
Dlg5
Tnfrsf13c

Vsir
Sash3
Lmbr1l
Clec4g
Dock8
Ccdc88b
Selenok
Il23a
Dnaja3
Clec2i
Gpnmb
Dock2
Tnfrsf21
Cd276
Scrib
Nckap1l
Card11
Lmo1
Crip3
Pawr
Cd209c
Cd209e
Cd209a
Glmn
Havcr2
Ripk2
Ripor2
Btla
Cblb
Il20rb
Tmem131l
Tnfrsf14

Armc5
Carmil2
Zc3h12d
Btn2a2
Muc19
Malt1
Tnfsf18
Vtcn1
Pde5a
Tarm1
Il27
Alkbh5
Vsig4
Rc3h2
Rasal3
Dhps
Cd59b
Rc3h1
Itgad
Lrrc32
Btnl2

Supplemental Table 4. Tissue residency memory core residency signatures derived from Mackay et al (*Science. 2016;352(6284):459-63.*)

Insig1
Haspin
Ddx3x
Dhcr24
Ppp1r16b
Klf6
Btg2
Cxcr6
Fosb
Jun
Hspa5
Nedd4
Plk3
Stard4
Tnfaip3
B4galnt4
Cd244a
Zfp683
Irf4
Cish
Mapkapk3
Sik1
Pygl
Ctnna1
Odc1
Per1
Dusp1
Atf3
Ldlrad4
Msmo1

Junb
Adgrg1
Nfkbid
Fosl2
Rgs2
Nr4a2
Dgat1
Arrdc3
Frmd4b
Nr4a1
Gpr171
Smim3
Eya2
Gpr55
Jaml
Ly6g5b
Csrnp1
Traf4
Zfp36
Glrx
Dusp5
Litaf
Gadd45b
Ifng
Osgin1
Abi3
Fam234b
Egr1
Hilpda
Skil
Rnf149
Hmgcs1

P4hb
Pnrc1
Gpr34
Ppp1r15a
Itgae
P2ry10
Ehd1
Dusp6
Xcl1
Spsb1
Isg20
Inpp4b
Neurl3
Hpgds
Rhob
Vdac1
Lad1
Fos
Cdh1
Cd69
Qpct
Hspd1

Supplemental Table 5. Gene symbols of T cell migration gene ontology term (GO:0072678)

Abl1
Abl2
Adam10
Adam17
Adam8
Aif1
Aire
Apod
App
Rhoa
Ccr6
Cd69
Coro1a
Cxcr3
Ccr2
Ccr7
Crk
Crkl
Ecm1
S1pr1
Fadd
Fut7
Gnai1
Icam1
Cxcl10
Itga4
Itgal
Itgb3
Itgb7
F11r
Lgals9

Xcl1
Ascl2
Cd200
Msn
Plec
Ccl21a
Ccl2
Ccl20
Ccl3
Ccl5
Cxcl12
Slc12a2
Spn
Tnfsf4
Wnt5a
Tnfsf14
Il27ra
Stk39
Cxcl13
Cxcl11
Ripk3
Cd200r1
Cxcl16
Pycard
Gpr15lg
Med23
Gpr15
Dock8
Selenok
Oxsr1
Cd99l2
Ripor2

Spns2
Tnfrsf14
Wnk1
Myo1g
Gpr183
Tmem102
Lrch1
Ccl26
Cd99
